# The m6A/m5C/m1A Regulated Gene Signature Predicts the Prognosis and Correlates With the Immune Status of Hepatocellular Carcinoma

**DOI:** 10.3389/fimmu.2022.918140

**Published:** 2022-06-27

**Authors:** Dan Li, Kai Li, Wei Zhang, Kong-Wu Yang, De-An Mu, Guo-Jun Jiang, Rong-Shu Shi, Di Ke

**Affiliations:** ^1^ Department of General Medicine, Affiliated Hospital of Zunyi Medical University, Zunyi, China; ^2^ Department of Oncology, Huanggang Central Hospital, Huanggang, China; ^3^ Department of Hepatobiliary and Pancreatic Surgery, The People’s Hospital of Jianyang City, Jianyang, China; ^4^ Department of Radiology, Affiliated Hospital of Zunyi Medical University, Zunyi, China

**Keywords:** m6A, m5C, m1A, HCC, prognosis

## Abstract

RNA modification of m6A/m5C/m1A contributes to the occurrence and development of cancer. Consequently, this study aimed to investigate the functions of m6A/m5C/m1A regulated genes in the prognosis and immune microenvironment of hepatocellular carcinoma (HCC). The expression levels of 45 m6A/m5C/m1A regulated genes in HCC tissues were determined. The functional mechanisms and protein–protein interaction network of m6A/m5C/m1A regulated genes were investigated. The Cancer Genome Atlas (TCGA) HCC gene set was categorized based on 45 m6A/m5C/m1A regulated genes, and survival analysis was used to determine the relationship between the overall survival of HCC patients in subgroups. Cox and least absolute shrinkage and selection operator (LASSO) regression analyses were used to construct the risk model and nomogram for m6A/m5C/m1A regulated genes. The relationships between m6A/m5C/m1A regulated gene subsets and risk model and immune cell infiltration were analyzed using CIBERSORT. m6A/m5C/m1A regulated genes were involved in mRNA and RNA modifications, mRNA and RNA methylation, mRNA and RNA stability, and other processes. There was a statistically significant difference between cluster1 and cluster2 groups of genes regulated by m6A/m5C/m1A. The prognosis of cluster1 patients was significantly better than that of cluster2 patients. There were statistically significant differences between the two cluster groups in terms of fustat status, grade, clinical stage, and T stage of HCC patients. The risk model comprised the overexpression of YBX1, ZC3H13, YTHDF1, TRMT10C, YTHDF2, RRP8, TRMT6, LRPPRC, and IGF2BP3, which contributed to the poor prognosis of HCC patients. The high-risk score was associated with prognosis, fustat status, grade, clinical stage, T stage, and M stage and was an independent risk factor for poor prognosis in HCC patients. High-risk score mechanisms included spliceosome, RNA degradation, and DNA replication, among others, and high-risk was closely related to stromal score, CD4 memory resting T cells, M0 macrophages, M1 macrophages, resting mast cells, CD4 memory activated T cells, and follicular helper T cells. In conclusion, the cluster subgroup and risk model of m6A/m5C/m1A regulated genes were associated with the poor prognosis and immune microenvironment in HCC and are expected to be the new tools for assessing the prognosis of HCC patients.

## Introduction

Liver cancer is one of the most prevalent cancers worldwide. Hepatocellular carcinoma (HCC) accounts for approximately 80% of liver cancer (1, 2). Recent studies have demonstrated that targeted therapy and immunotherapy significantly affect HCC patient survival (3–6). For example, Shimose et al. reported that sorafenib (SORA) improves overall survival (OS) in HCC patients. SORA can improve the prognosis of patients with unresectable HCC as first-line therapy (4). Nonetheless, the prognosis for HCC patients remains unsatisfactory. Therefore, it is extremely important to identify new targets or immunotherapies to improve the prognosis of HCC patients.

In eukaryotic messenger RNA (mRNA) regulation, *N* ^6^-methyladenosine (m6A), *N* ^1^-methyladenosine (m1A), and 5-methylcytosine (m5C) modifications exist. Several studies have confirmed that m6A, m1A, and m5C regulated genes play important roles in m6A, m1A, and m5C modifications (7–11). Recent research has found that m6A, m1A, and m5C regulated gene expression levels are associated with tumor progression (11–16). For example, the m6A-regulated gene methyltransferase 3 (METTL3), METTL14, and WTAP are involved in the initiation of the m6A modification process. METTL3 expression is elevated in endometrioid epithelial ovarian cancer (EEOC) tissues. The METTL3 overexpression levels correlate with the degree of malignancy and the OS of EEOC patients. Inhibiting METTL3 expression in TOV-112D and CRL-11731D cells attenuates cancer cell proliferation and migration and promotes apoptosis. METTL3 overexpression promotes EEOC progression by regulating m6A methylation (11). The m5C methyltransferase NSUN2 is significantly upregulated in gastric cancer and is predictive of a poor prognosis in gastric cancer patients. *In vitro*, NSUN2 promotes the proliferation, migration, and invasion of gastric cancer cells. Small ubiquitin-like modifier (SUMO)-2/3 promotes the oncogenic activity of NSUN2 by stabilizing NSUN2 (15). The risk model has been employed as a tool for determining the prognosis of cancer patients (17, 18). Currently, the roles of m6A, m1A, and m5C regulated genes in HCC progression are not yet fully understood. Therefore, in this study, data from The Cancer Genome Atlas (TCGA) database were used to elucidate the biological functions and network involved in m6A/m1A/m5C regulated genes (8), and the roles of m6A/m1A/m5C regulated genes in the prognosis of HCC patients were identified. The risk model and nomogram of m6A/m1A/m5C regulated genes were constructed to determine the prognosis of HCC patients.

## Materials and Methods

### The Expression Levels of m6A/m1A/m5C Regulated Genes

Gene expression data of HCC patients were extracted from TCGA database. A total of 50 normal liver tissue samples and 374 HCC tissue samples were included. The Writer, Reader, and Eraser genes of m6A/m1A/m5C were obtained from the literature (8), and the obtained m6A, m1A, and m5C regulated genes were merged and de-duplicated. Subsequently, the m6A/m1A/m5C regulated gene expression data were retrieved in normal and HCC tissues, and the expression levels of m6A/m1A/m5C regulated genes in HCC tissues were analyzed using the Limma package.

### The Relationship Between m6A/m1A/m5C Regulated Genes

The network of 45 m6A/m1A/m5C regulated genes in the STRING database was explored, and Cytoscape (version: 3.8.2) software was used to show the relationship between the protein–protein interaction (PPI) network of m6A/m1A/m5C regulated genes.

### Gene Ontology and Kyoto Encyclopedia of Genes and Genomes Analyses

Gene Ontology (GO) and Kyoto Encyclopedia of Genes and Genomes (KEGG) methods are frequently used to assess the biological functions and the signaling mechanisms of polygenes (19). In this study, GO and KEGG methods were used to investigate the biological functions and signaling mechanisms associated with the 45 m6A/m1A/m5C regulated genes. In addition, the biological functions and signaling mechanisms involved in the abnormally expressed genes related to the 45 m6A/m1A/m5C regulated genes were determined using GO and KEGG methods.

### Identification of m6A/m1A/m5C Regulated Gene Subgroups Using Consensus Clustering

The “ConsensusClusterPlus” package was used to cluster 45 m6A/m1A/m5C regulated genes in 374 HCC tissues extracted from TCGA database into different subtypes. TCGA 374 HCC tissues were divided into cluster1 and cluster2 groups based on the optimal k value. Principal component analysis (PCA) was used to determine the differences between the two groups of patients. The Kaplan–Meier (K-M) survival analysis and log-rank test were used to assess the relationship between survival and clinicopathological characteristics of HCC patients in the two cluster groups.

### Cluster-Related Differentially Expressed Genes Based on the m6A/m1A/m5C Regulated Genes

Cluster1 and cluster2 groups of the m6A/m1A/m5C regulated gene subsets were matched with the tissue samples from 374 HCC patients, and the Limma package was used to determine the differentially expressed genes (DEGs) in cluster1 and cluster2 groups. The false discovery rate (FDR) <0.05 and log fold change (FC) = 2 were used to identify DEGs associated with m6A/m1A/m5C regulated gene subsets.

### Identification of Roles m6A/m1A/m5C Regulated Gene Subgroups Associated With Differentially Expressed Genes

The relationship between the m6A/m1A/m5C regulated gene subgroups related to DEGs and the prognosis of HCC patients was using univariate Cox regression analysis (p < 0.001). Subsequently, the Limma package was used to investigate the expression levels of DEGs that were modulated by m6A/m1A/m5C in subgroups of genes associated with prognosis in 50 normal liver tissue samples and 374 HCC tissue samples.

### Cluster-Related Genes Based on the m6A/m1A/m5C Regulated Gene Subgroups Using Consensus Clustering

The expression data of 37 prognosis related DEGs in 374 HCC tissues were extracted. The optimal k value was determined by consensus clustering analysis, and the 374 HCC tissues from TCGA were grouped. PCA was used to determine the differences between subgroups of HCC patients. The K-M survival analysis and log-rank test were used to determine the relationship between prognostic and clinicopathological factors in patient subgroups.

### Construction of a Risk Model and Nomograms Associated With m6A/m1A/m5C Regulated Genes

The expression data of 45 m6A/m1A/m5C regulated genes were matched and merged with the prognosis data of HCC patients, and the relationship between m6A/m1A/m5C regulated genes and the prognosis of HCC patients was investigated using univariate Cox regression analysis (p < 0.05). The least absolute shrinkage and selection operator (LASSO) regression analysis was used to construct a risk model for m6A/m1A/m5C regulated genes, and the risk score for m6A/m1A/m5C regulated genes was calculated as follows: risk score = Σ (ExpmRNAn × βmRNAn) (20). Correlation analysis explored the relationship between the risk score and the expression levels of model factors, including YBX1, ZC3H13, YTHDF1, TRMT10C, YTHDF2, RRP8, TRMT6, LRPPRC, and IGF2BP3. The K-M survival analysis was performed to determine the prognosis of HCC patients in the high- and low-risk groups, as well as the construction of nomogram related to m6A/m1A/m5C regulated genes.

### Identification of m6A/m1A/m5C Regulated Gene Expression in Hepatocellular Carcinoma Tissues

Cancer and normal liver tissues of 12 HCC patients diagnosed through pathology at our hospital were collected between February and April 2022. All 12 HCC patients signed the informed consent, which was reviewed and approved by the ethics committee of our hospital. With the use of a standard qRT-PCR protocol, the expression levels of YBX1, ZC3H13, YTHDF1, TRMT10C, YTHDF2, RRP8, TRMT6, LRPPRC, and IGF2BP3 in 12 HCC tissues and normal liver tissues were determined (21). The primer numbers for TRMT10C and RRP8 that were purchased from GeneCopoeia (Guangzhou, China) were HQP013870 and HQP097040, respectively. **Table 1** shows the primer sequences for the m6A/m1A/m5C regulated genes YBX1, ZC3H13, YTHDF1, YTHDF2, TRMT6, LRPPRC, and IGF2BP3.

**Table 1:** The primer sequences of m6A/m1A/m5C regulated genes.

Gene	Sequence (5′–3′)	
	Forward	Reverse
YBX1	GGTCCTCCACGCAATTACCA	GTTGTCAGCACCCTCCATCA
ZC3H13	AAAGGAGGGTTTCACCAGAAGTG	CGCTTCGGAGATTTGCTAGAC
YTHDF1	ACCTGTCCAGCTATTACCCG	TGGTGAGGTATGGAATCGGAG
YTHDF2	TAGCCAACTGCGACACATTC	CACGACCTTGACGTTCCTTT
TRMT6	GGTGCTGAAACGTGAAGATGT	CTTGGGCTGTAGACTTCCTCC
LRPPRC	TTCAGTGCTCTCGTCACAGG	GTCGCGGTCCATGAAGTAAT
IGF2BP3	AGTTGTTGTCCCTCGTGACC	GTCCACTTTGCAGAGCCTTC

### Clinical Values of a Risk Model Associated With m6A/m1A/m5C Regulated Genes

The relationship between the high- and low-risk groups and clinicopathological characteristics of HCC patients were assessed using the log-rank test. To understand the clinical value of the risk model, univariate and multivariate Cox regression analyses were performed to establish the relationship between age, gender, grade, clinical stage, T stage, M stage, N stage, and risk score value and OS of HCC patients.

### Signaling Mechanisms of the Risk Model

Gene Set Enrichment Analysis (GSEA) is commonly used to investigate the signaling mechanisms of risk model (22). HCC patients were divided into the high- and low-risk groups based on the median risk score. With the use of GSEA (version 4.1.0), the effect of the risk model constructed by m6A/m1A/m5C regulated genes on each gene set was determined. GSEA was run for 1,000 cycles (Nominal p < 0.05).

### The Relationship Between the Constructed Model and Immune Infiltrating Cells Based on the m6A/m1A/m5C Regulated Genes

The expression levels of immune cells in the 374 HCC tissues were calculated using the CIBERSORT method. The immune cells of 374 HCC patients were divided into two groups: cluster1 and cluster2 groups. A t-test was used to determine whether there was a difference in the expression levels of HCC immune cells in cluster1 and cluster2 groups. The high- and low-risk models constructed based on the m6A/m1A/m5C regulated genes were combined with the immune cell data of 374 HCC patients, and the expression levels of immune cells in the high- and low-risk groups were explored. In addition, correlation analysis investigated the relationship between the risk score and immune cell infiltration in HCC.

### The Relationship Between the Constructed Risk Model of m6A/m1A/m5C Regulated Genes and Immune Cell Markers

The expression levels of immune cell markers extracted from 374 HCC tissues in TCGA database were acquired. The risk scores were combined with the immune cell marker gene data of cancer patients, and the expression levels of immune cell markers in the high- and low-risk groups were explored using the t-test. In addition, correlation analysis explored the relationship between the risk scores and immune-infiltrating cell markers in HCC tissues.

### Statistical Analysis

The expression levels of m6A/m1A/m5C regulated genes and m6A/m1A/m5C regulated gene subgroup-related genes in HCC tissues were identified using the Limma package. Cox, LASSO, and K-M survival analyses were used to identify m6A/m1A/m5C regulated genes associated with the prognosis of HCC patients. The relationship between models based on m6A/m1A/m5C regulated genes and immune cell infiltration was determined using correlation analysis (p < 0.05).

## Results

### Identification of Expression Levels of m6A/m1A/m5C Regulated Genes in Hepatocellular Carcinoma Tissues

**Figure 1:**
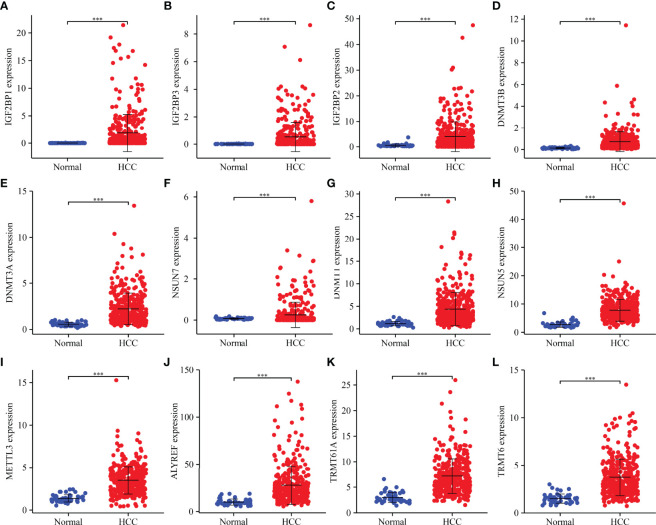
m6A/m1A/m5C regulated DEGs in HCC tissues as determined based on fold change. **(A)** IGF2BP1; **(B)** IGF2BP3; **(C)** IGF2BP2; **(D)** DNMT3B; **(E)** DNMT3A; **(F)** NSUN7; **(G)** DNMT1; **(H)** NSUN5; **(I)** METTL3; **(J)** ALYREF; **(K)** TRMT61A; **(L)** TRMT6. DEGs, differentially expressed genes; HCC, hepatocellular carcinoma; ***P < 0.001.

Compared to normal liver tissues, abnormal expression levels of m6A/m1A/m5C regulated genes were observed in HCC tissues (**Table 2**). The heatmap depicts the expression levels of m6A/m1A/m5C regulated genes in HCC tissues (**Figure S1**). In HCC tissues, 41 (91.11%) of the m6A/m1A/m5C regulated genes were statistically significant. Based on the fold change, the expression levels of 12 m6A/m1A/m5C regulated genes in HCC tissues are shown (**Figure 1**). IGF2BP1, IGF2BP3, IGF2BP2, DNMT3B, DNMT3A, DNMT1, NSUN7, NSUN5, ALYREF, METTL3, TRMT6, and TRMT61A were overexpressed in HCC tissues relative to normal tissues.

**Table 2:** The expression levels of m6A/m1A/m5C regulated genes in HCC tissues.

Gene	Normal	HCC	p	Gene	Normal	HCC	p
NSUN3	0.655	0.969	5.19E−09	DNMT3B	0.128	0.716	7.59E−21
NSUN4	1.289	2.019	6.67E−12	DNMT1	1.169	4.380	7.50E−21
HNRNPC	19.336	34.922	1.48E−23	IGF2BP2	0.520	3.983	1.22E−07
ALKBH3	3.363	4.492	9.79E−05	BMT2	0.465	0.984	7.46E−15
WTAP	5.134	7.269	1.39E−06	DNMT3A	0.555	2.215	7.94E−24
METTL14	2.266	2.585	0.061882534	YTHDF2	8.640	11.803	2.46E−09
YBX1	78.054	136.118	5.80E−16	RBM15B	3.371	6.805	6.26E−23
TRMT61B	2.115	3.543	2.03E−11	NSUN7	0.071	0.253	6.30E−05
KIAA1429	2.038	4.790	5.59E−24	YTHDC1	4.252	5.545	3.13E−05
RBM15	1.473	1.978	3.27E−06	METTL3	1.366	3.488	7.37E−24
TET2	0.389	0.466	0.524844577	YTHDC2	1.568	2.150	1.38E−05
TRDMT1	0.097	0.147	0.052573721	RBMX	6.072	13.653	7.39E−25
ALKBH1	1.814	2.386	6.76E−08	NOP2	3.073	6.927	1.01E−19
NSUN5	2.718	7.806	9.19E−27	RRP8	1.877	2.739	1.58E−14
HNRNPA2B1	35.391	65.222	4.02E−23	TRMT6	1.496	3.726	6.90E−23
IGF2BP1	0.009	1.858	2.86E−16	ALKBH5	18.683	23.697	0.00087808
FMR1	3.610	6.177	1.14E−10	LRPPRC	7.691	13.042	4.68E−15
RBMY1A1	0.000	0.002	0.045862588	IGF2BP3	0.010	0.528	1.66E−12
METTL16	0.959	1.654	4.25E−12	NSUN2	7.100	10.449	3.25E−12
ZC3H13	3.471	3.829	0.831175703	FTO	1.372	1.921	2.86E−05
TRMT61A	2.981	7.168	2.85E−23	ALYREF	9.908	27.733	2.33E−21
YTHDF1	9.061	15.309	1.79E−20	YTHDF3	7.164	10.615	9.66E−09
TRMT10C	7.618	11.171	2.92E−12				

HCC, hepatocellular carcinoma.

### The Functional Mechanisms and Roles of m6A/m1A/m5C Regulated Genes

GO annotation revealed that the m6A/m1A/m5C regulated genes participate in RNA modification and methylation, macromolecule methylation, mRNA methylation and modification, RNA regulation and mRNA stability, ncRNA processing, regulation of mRNA catabolic process, RNA splicing, mRNA catabolic process, mRNA transport, and other processes (**Table S1**). KEGG analysis involved the microRNAs in cancer, spliceosome, and RNA transport. **Figure S2** showed the PPI network between m6A/m1A/m5C regulated genes.

### Evaluation of Clinical Values of m6A/m1A/m5C Regulated Gene Subgroups in Hepatocellular Carcinoma Patients Using Consensus Clustering

**Figure 2:**
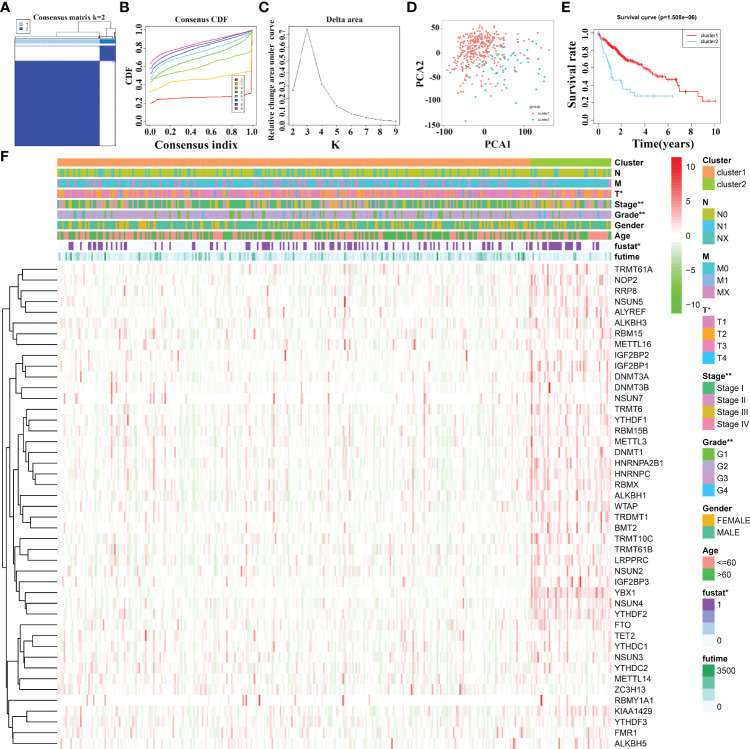
The clinical values in m6A/m1A/m5C regulated gene subgroups in HCC patients based on consensus clustering. **(A–C)** The consensus clustering of m6A/m1A/m5C regulated genes. **(D)** PCA. **(E)** K-M survival analysis. **(F)** The relationships between the clinicopathological features and m6A/m1A/m5C regulated gene subgroups. HCC, hepatocellular carcinoma; PCA, principal component analysis; K-M, Kaplan–Meier; *P < 0.05; **P < 0.01.

Based on 45 m6A/m1A/m5C regulated gene expression data, and the optimal k value of 2, 374 HCC patients are divided into cluster1 and cluster2 groups (**Figures 2A–C**). PCA revealed significant differences between cluster1 and cluster2 groups (**Figure 2D**). The K-M survival analysis demonstrated that the OS of patients in cluster1 was significantly better than that of patients in cluster2 (**Figure 2E**). In addition, there was a statistically significant difference between HCC patients in the two cluster groups in terms of clinical stage, T stage, tumor grade, and fustat status (**Figure 2F**).

### The Functions and Signaling Pathways of Cluster-Related Differentially Expressed Genes Based on the m6A/m1A/m5C Regulated Genes

**Figure 3:**
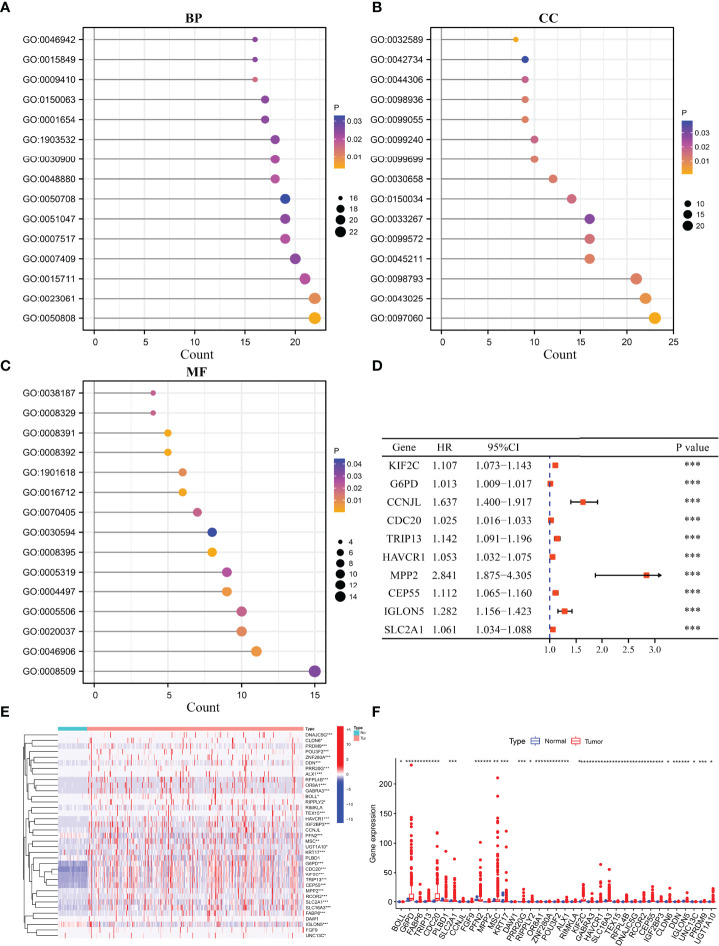
The expression levels and roles of cluster-related DEGs. **(A–C)** The biological functions of cluster-related DEGs. **(D)** The prognostic value of cluster-related genes as determined using the Cox analysis. **(E, F)** The expression of cluster-related genes. BP, biological process; CC, cellular component; MF, molecular function; DEGs, differentially expressed genes; *P < 0.05; **P < 0.01; ***P < 0.001.

Compared to cluster1 group, cluster2 group HCC tissues displayed 371 cluster-related DEGs (**Table S2**), including 315 overexpressed genes and 56 downregulated genes. **Figure S3** shows the top 20 cluster-related DEGs in HCC tissues. The m6A/m1A/m5C regulated gene subsets related DEGs were found to be involved in signaling release, neurotransmitter transport, drug catabolic process, positive regulation of protein secretion, regulation of secretion, calcium ion regulated exocytosis, hormone transport, regulation of protein secretion, and others using GO analysis (**Figures 3A–C**) and involved in bile secretion, neuroactive ligand–receptor interaction, and PPAR signaling pathway using KEGG analysis.

### Prognostic Values ​​and Expression Levels of Cluster-Related Differentially Expressed Genes

Univariate Cox regression analysis revealed that 127 DEGs were associated with poor prognosis in HCC patients based on the p < 0.05 (**Table S3**), and 37 DEGs were associated with poor prognosis in HCC patients based on the p < 0.001 (**Table 3**). The 37 DEGs were KIF2C, G6PD, CCNJL, CDC20, TRIP13, HAVCR1, MPP2, CEP55, IGLON5, SLC2A1, ALX1, TEX15, MSC, IGF2BP3, FGF9, OR8A1, RCOR2, PFN2, DDN, CLDN6, UGT1A10, ZNF280A, PRR20G, DNAJC5G, SLC16A3, BOLL, KRT17, DAW1, RFPL4B, RIPPLY2, UNC13C, RIMKLA, GABRA3, PLBD1, PRDM9, POU3F2, and FABP6 (**Figure 3D** and **Table 3**). **Figures 3E, F** showed the expression levels of KIF2C, G6PD, CCNJL, CDC20, TRIP13, HAVCR1, MPP2, CEP55, IGLON5, SLC2A1, ALX1, TEX15, MSC, IGF2BP3, FGF9, OR8A1, RCOR2, PFN2, DDN, CLDN6, UGT1A10, ZNF280A, PRR20G, DNAJC5G, SLC16A3, BOLL, KRT17, DAW1, RFPL4B, RIPPLY2, UNC13C, RIMKLA, GABRA3, PLBD1, PRDM9, POU3F2, and FABP6 in HCC tissues.

**Table 3:** Association of the 37 cluster-related DEGs with the prognosis of HCC patients.

Gene	HR	HR.95L	HR.95H	p
BOLL	66,403.61842	222.1987651	19,844,577.16	0.000134526
G6PD	1.012815378	1.008712945	1.016934494	7.79E−10
FABP6	1.087079254	1.03484665	1.141948234	0.000889414
TRIP13	1.142197446	1.090903993	1.195902678	1.42E−08
CDC20	1.024872506	1.016463812	1.033350761	5.07E−09
PLBD1	1.026843644	1.011167244	1.042763079	0.00073873
SLC2A1	1.060677084	1.033841048	1.088209719	6.63E−06
CCNJL	1.636945867	1.39815106	1.916525223	9.02E−10
FGF9	11.85093328	3.70028856	37.95504523	3.14E−05
PFN2	1.02363973	1.012006001	1.035407197	6.16E−05
MPP2	2.840864973	1.874873886	4.304563552	8.46E−07
MSC	1.010073927	1.005388429	1.014781261	2.39E−05
KRT17	1.023577027	1.011335869	1.035966351	0.0001469
DAW1	2,119.15023	39.91103009	112,520.2152	0.000157415
PRR20G	1.183500918	1.086811403	1.288792534	0.000106897
RIPPLY2	1.27895766	1.115462862	1.466416096	0.000422259
OR8A1	85.06178746	9.973131929	725.5000473	4.85E−05
ZNF280A	11.21303049	3.306991907	38.02006666	0.000104542
POU3F2	2.431600762	1.441817888	4.100852345	0.000861814
ALX1	1.555968662	1.279752059	1.891802759	9.26E−06
RIMKLA	2.302992438	1.436296604	3.692673336	0.00053421
KIF2C	1.107275694	1.072814998	1.142843327	2.67E−10
GABRA3	1.199491202	1.081139725	1.330798519	0.000599349
HAVCR1	1.0533519	1.032254557	1.074880433	4.77E−07
SLC16A3	1.048105558	1.023234862	1.07358076	0.000125798
TEX15	3.351249687	1.951601305	5.754697147	1.17E−05
RFPL4B	1.165840648	1.070643844	1.269501919	0.000414669
DNAJC5G	3.118040294	1.745745056	5.569069344	0.000121667
RCOR2	1.188220977	1.092485363	1.292346001	5.73E−05
CEP55	1.111571561	1.065462685	1.15967584	9.91E−07
IGF2BP3	1.318404695	1.158327301	1.500604311	2.85E−05
CLDN6	1.14192628	1.069149958	1.219656436	7.81E−05
DDN	9.859609362	3.215354399	30.23364915	6.26E−05
IGLON5	1.282484083	1.156020228	1.422782563	2.64E−06
UNC13C	4,236.152744	37.83758883	474,263.5729	0.000521844
PRDM9	3.179935821	1.611894472	6.273358462	0.000846528
UGT1A10	1.130090713	1.06349902	1.200852089	7.92E−05

DEGs, differentially expressed genes; HCC, hepatocellular carcinoma.

### The Effect of 37 Cluster-Related Differentially Expressed Gene Subgroups on the Prognosis of Hepatocellular Carcinoma Patients Using Consensus Clustering

**Figure 4:**
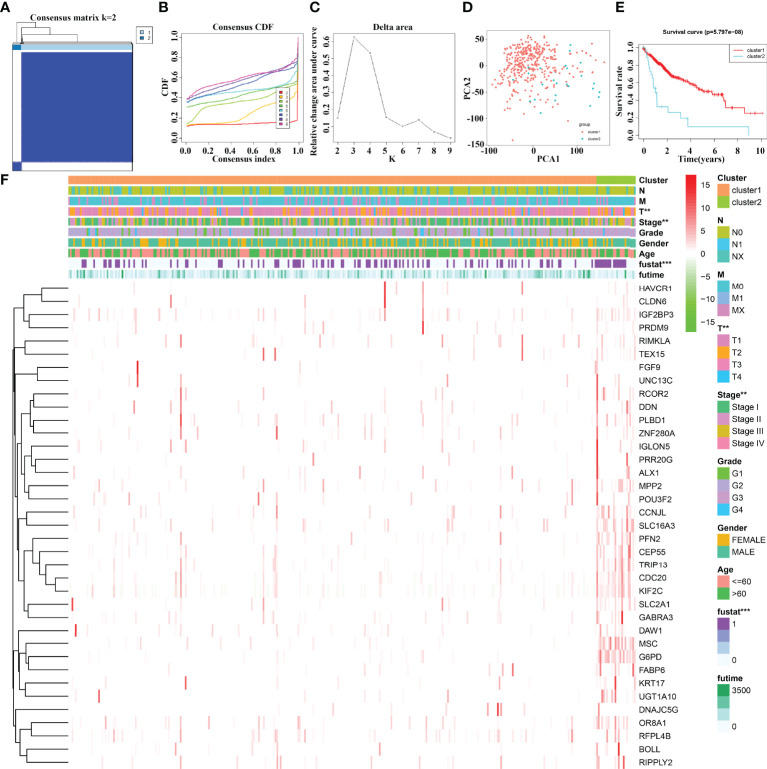
The prognostic value of 37 cluster-related DEG subgroups in HCC based on consensus clustering. **(A–C)** The consensus clustering of 37 cluster-related DEGs. **(D)** PCA. **(E)** K-M survival analysis. **(F)** The relationships between the clinicopathological features and 37 cluster-related DEG subgroups. HCC, hepatocellular carcinoma; PCA, principal component analysis; K-M, Kaplan–Meier; differentially expressed genes; **P < 0.01; ***P < 0.001.

The 37 cluster-related differentially expressed gene subgroups divided the 374 HCC patients into cluster1 and cluster2 groups (**Figures 4A–C**). PCA showed significant differences between cluster1 and cluster2 groups (**Figure 4D**). The K-M survival analysis revealed that the OS of HCC patients in cluster1 group was significantly better than that of the patients in cluster2 group (**Figure 4E**). There are statistically significant differences between HCC patients in cluster1 and cluster2 groups in terms of clinical stage, T stage, and fustat status (**Figure 4F**).

### Construction of m6A/m1A/m5C Regulated Gene Related Risk Model and Nomogram

**Figure 5:**
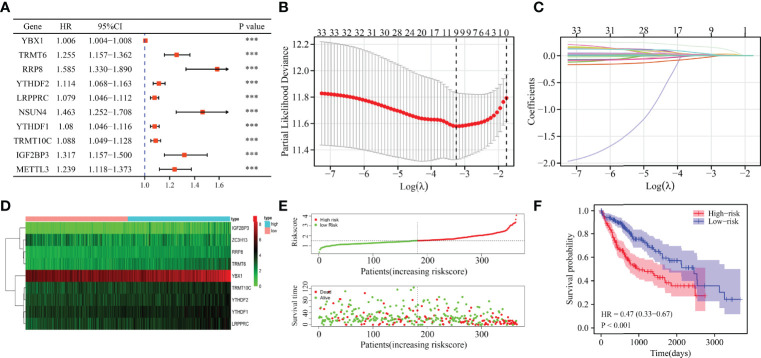
The risk model constructed using the LASSO method for predicting the prognosis of patients. **(A)** Cox method showing the prognostic value of m6A/m1A/m5C regulated genes. **(B, C)** The 9 m6A/m5C/m1A-regulated genes identified using least absolute shrinkage and LASSO method. **(D–F)** The association of high-risk score with the m6A/m1A/m5C regulated genes and prognosis of HCC patients. HCC, hepatocellular carcinoma; LASSO, least absolute shrinkage and selection operator; ***P < 0.001.

**Figure 6:**
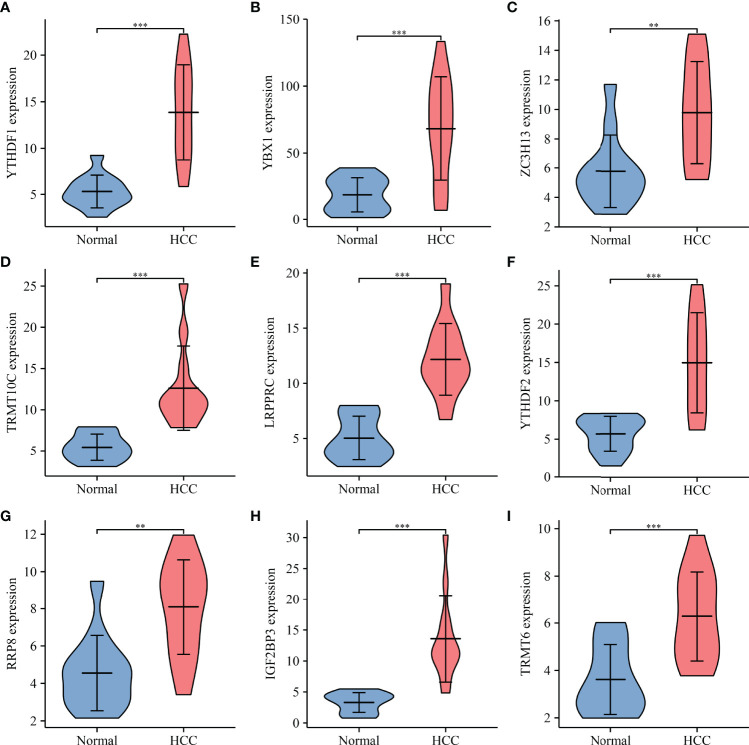
The expression profile of risk model genes in HCC tissues. **(A)** YTHDF1; **(B)** YBX1; **(C)** ZC3H13; **(D)** TRMT10C; **(E)** LRPPRC; **(F)** YTHDF2; **(G)** RRP8; **(H)** IGF2BP3; **(I)** TRMT6. HCC, hepatocellular carcinoma; **P < 0.01; ***P < 0.001.

**Figure 7:**
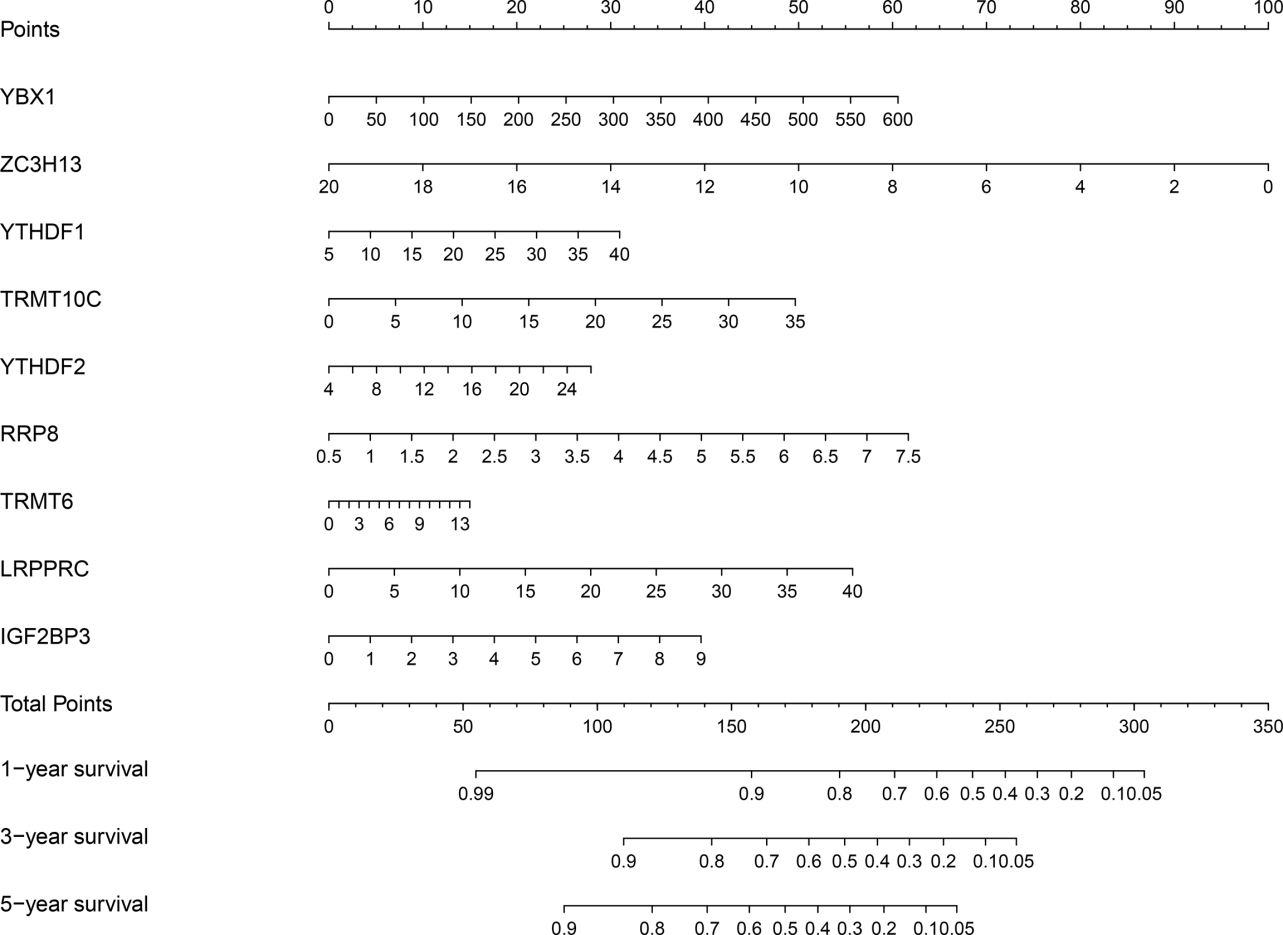
Prognostic nomogram based on the 9 m6A/m5C/m1A-regulated genes in HCC patients. HCC, hepatocellular carcinoma.

Univariate Cox regression analysis revealed that YBX1, TRMT6, RRP8, YTHDF2, LRPPRC, NSUN4, YTHDF1, TRMT10C, IGF2BP3, METTL3, DNMT3A, DNMT1, NOP2, TRMT61B, RBM15B, KIAA1429, ALYREF, HNRNPA2B1, TRMT61A, ALKBH1, HNRNPC, RBMX, DNMT3B, BMT2, WTAP, NSUN3, TRDMT1, NSUN2, NSUN5, ZC3H13, IGF2BP1, YTHDC1, and IGF2BP2 were influencing factors for poor prognosis in HCC patients (**Table 4** and **Figure 5A**). The minimum λ value was determined using the LASSO algorithm, and the risk model feature was constructed based on the 9 m6A/m1A/m5C regulated genes (**Figures 5B, C**). **Figure 5D** shows the relationship between the YBX1, ZC3H13, YTHDF1, TRMT10C, YTHDF2, RRP8, TRMT6, LRPPRC, and IGF2BP3 expression levels in the high- and low-risk groups. Furthermore, significant correlations between the YBX1, ZC3H13, YTHDF1, TRMT10C, YTHDF2, RRP8, TRMT6, LRPPRC, and IGF2BP3 expression levels and the risk score were reported (**Figure S4**). **Figure 5E** shows the relationship between the risk score and survival time of HCC patients. In the risk model based on expression levels of YBX1, ZC3H13, YTHDF1, TRMT10C, YTHDF2, RRP8, TRMT6, LRPPRC, and IGF2BP3, the K-M survival analysis revealed a poor prognosis in high-risk HCC patients (**Figure 5F**). Furthermore, the HCC tissues from our hospital showed high expression levels of YBX1, ZC3H13, YTHDF1, TRMT10C, YTHDF2, RRP8, TRMT6, LRPPRC, and IGF2BP3 (**Figure 6**). The gene expression trends in TCGA database were consistent with the results of gene expression in our hospital tissues. Therefore, a nomogram incorporating the m6A/m1A/m5C regulated genes YBX1, ZC3H13, YTHDF1, TRMT10C, YTHDF2, RRP8, TRMT6, LRPPRC, and IGF2BP3 was constructed (**Figure 7**).

**Table 4:** The effect of m6A/m1A/m5C regulated genes on the prognosis of HCC patients determined using univariate Cox regression analysis.

Gene	HR	HR.95L	HR.95H	p
YBX1	1.005985366	1.004264511	1.007709171	8.40E−12
TRMT6	1.255138452	1.156845284	1.361783252	4.72E−08
RRP8	1.585178413	1.329868826	1.889502597	2.72E−07
YTHDF2	1.114292914	1.068070777	1.162515372	5.54E−07
LRPPRC	1.07859041	1.046145941	1.112041091	1.20E−06
NSUN4	1.462794699	1.252448731	1.708467803	1.57E−06
YTHDF1	1.080422184	1.04609785	1.11587276	2.65E−06
TRMT10C	1.087741415	1.04898593	1.127928748	5.53E−06
IGF2BP3	1.317391952	1.157171249	1.499796644	3.10E−05
METTL3	1.238985364	1.117920952	1.373160357	4.41E−05
DNMT3A	1.200194861	1.096206398	1.314047891	7.93E−05
DNMT1	1.071270978	1.033680613	1.110228337	0.000158354
NOP2	1.093983058	1.043957268	1.146406054	0.000169028
TRMT61B	1.200458086	1.090448995	1.321565358	0.000194766
RBM15B	1.130717082	1.059753128	1.206432976	0.000203279
KIAA1429	1.157967533	1.070574195	1.252494983	0.000249022
ALYREF	1.013042448	1.00598989	1.020144448	0.000277523
HNRNPA2B1	1.012503536	1.005358854	1.019698993	0.000583449
TRMT61A	1.077473783	1.032043335	1.124904074	0.000686328
ALKBH1	1.394863768	1.143761743	1.701092858	0.001014692
HNRNPC	1.023607287	1.009297907	1.03811954	0.001160338
RBMX	1.044664953	1.017243554	1.07282554	0.001283226
DNMT3B	1.232694499	1.084339494	1.401346844	0.001385893
BMT2	1.352050257	1.117650061	1.635610251	0.001902935
WTAP	1.088194245	1.031624448	1.147866083	0.001915462
NSUN3	1.726777348	1.182289002	2.522022961	0.0047078
TRDMT1	3.785635247	1.426675045	10.04505846	0.007502984
NSUN2	1.047056797	1.012225865	1.08308627	0.007722841
NSUN5	1.033657382	1.007547712	1.06044366	0.011212183
ZC3H13	0.909935462	0.838113494	0.987912199	0.024457281
IGF2BP1	1.054425564	1.006534019	1.10459582	0.025445348
YTHDC1	1.081213814	1.00738535	1.16045296	0.030473944
IGF2BP2	1.023648662	1.000388004	1.047450168	0.046256781

HCC, hepatocellular carcinoma.

### Clinical Values of the Risk Model Associated With the m6A/m1A/m5C Regulated Genes

**Figure 8:**
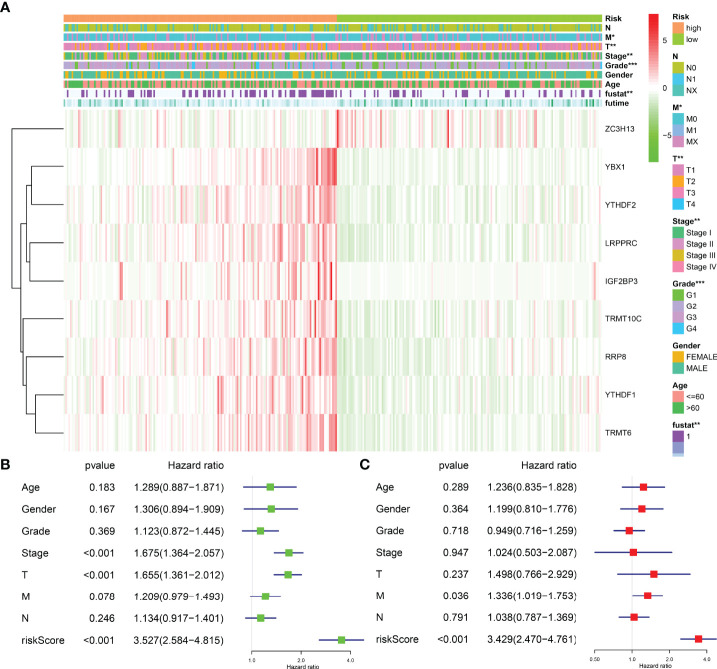
The correlation between risk model and the prognosis and clinicopathological characteristics of HCC patients. **(A)** The relationships between the clinicopathological features and risk model. **(B, C)** Univariate and multivariate Cox regression analyses for the prognostic factors of HCC patients. HCC, hepatocellular carcinoma; *P < 0.05; **P < 0.01; ***P < 0.001.

The risk model was related to the tumor grade, clinical stage, T stage, distant metastasis, and fustat status of HCC patients, according to an analysis of clinicopathological characteristics of HCC patients in the high- and low-risk groups (**Figure 8A**). Univariate Cox regression analysis revealed that the clinical stage, T stage, and risk score were risk factors for the dismal prognosis in HCC patients (**Figure 8B**). Multivariate Cox regression analysis revealed that distant metastasis and risk score were risk factors for poor prognosis in HCC patients (**Figure 8C**).

### Signaling Mechanisms Associated With a High-Risk Score

A high-risk score is associated with spliceosome regulation, cell cycle, base excision repair, RNA degradation, oocyte meiosis, ubiquitin-mediated proteolysis, pyrimidine metabolism, DNA replication, homologous recombination, mismatch repair, nucleotide excision repair, cytosolic DNA sensing pathway, endocytosis, neurotrophin signaling pathway, basal transcription factors, pathways in cancer, WNT signaling pathway, etc. (**Figure S5** and **Table 5**).

**Table 5:** Signaling mechanisms of high-risk score.

Name	Size	NES	NOM p
Spliceosome	126	1.991113	0
Cell cycle	124	1.9617648	0
RNA degradation	57	1.9323748	0
Oocyte meiosis	112	1.8970882	0
Ubiquitin-mediated proteolysis	133	1.8952316	0
Purine metabolism	156	1.7711903	0
Base excision repair	33	1.9551224	0.00204918
Homologous recombination	28	1.7728467	0.004016064
Pyrimidine metabolism	97	1.8858767	0.00409836
DNA replication	36	1.7922695	0.004132231
Endocytosis	181	1.6736333	0.004166667
Nucleotide excision repair	44	1.753949	0.006072875
Progesterone mediated oocyte maturation	85	1.6414596	0.009920635
Bladder cancer	42	1.6187055	0.010162601
Vasopressin-regulated water reabsorption	44	1.6811514	0.010204081
Neurotrophin signaling pathway	126	1.6216841	0.010351967
Mismatch repair	23	1.7572185	0.012072435
Cytosolic DNA sensing pathway	54	1.7055105	0.014056225
Pathways in cancer	325	1.5235739	0.01446281
Basal transcription factors	35	1.6122106	0.016393442
Thyroid cancer	29	1.6354401	0.02053388
Non-small cell lung cancer	54	1.5427107	0.020618556
Regulation of actin cytoskeleton	212	1.5155553	0.024539877
FC gamma R-mediated phagocytosis	95	1.5975801	0.024590164
N glycan biosynthesis	46	1.6555921	0.030487806
Pathogenic *Escherichia coli* infection	55	1.5665298	0.033333335
*Vibrio cholerae* infection	54	1.5166115	0.036511157
WNT signaling pathway	150	1.4821546	0.036511157
Pancreatic cancer	70	1.5321012	0.038617887
Epithelial cell signaling in *Helicobacter pylori* infection	68	1.5150603	0.039215688
Long-term potentiation	70	1.4539124	0.041749503

NES, normalized enrichment score; NOM, nominal.

### Immune Cell Infiltration in Hepatocellular Carcinoma Correlates With the Cluster Groups and Risk Model of m6A/m1A/m5C Regulated Genes

**Figure 9:**
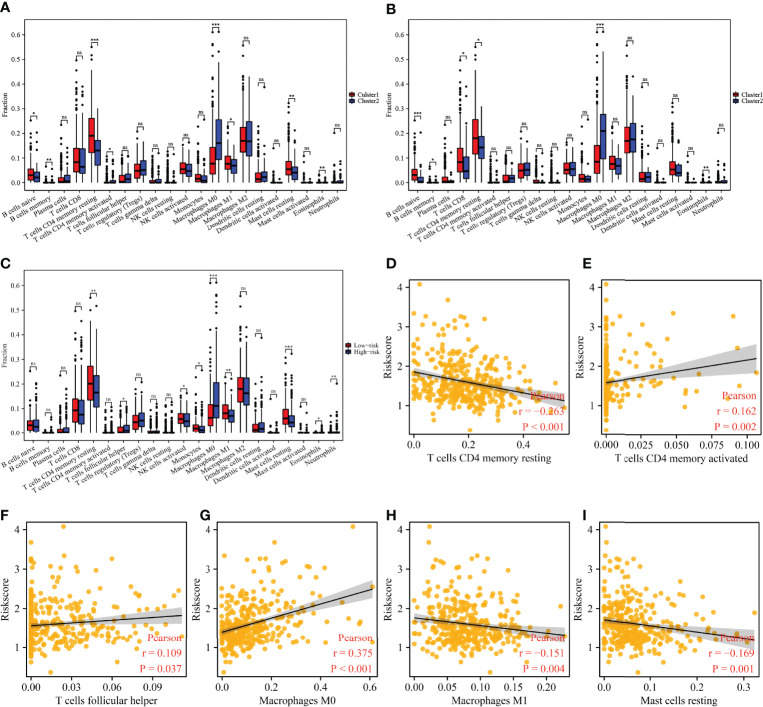
The correlation between the cluster groups and risk model and immune cell infiltration in HCC. **(A)** The expression levels of immune cells in m6A/m1A/m5C regulated gene subgroups. **(B)** The expression levels of immune cells in 37 cluster-related DEG subgroups. **(C)** The expression levels of immune cells in high- and low-risk groups. **(D–I)** Correlation between the risk model and immune cells. HCC, hepatocellular carcinoma; differentially expressed genes; ns, not statistically significant; *P < 0.05; **P < 0.01; ***P < 0.001.

There were significant differences in the expression levels of B cells naive, B cells memory, CD4 memory resting T cells, CD4 memory activated T cells, M0 macrophages, M1 macrophages, resting mast cells, and eosinophils among the 45 m6A/m1A/m5C regulated gene subgroups (**Figure 9A**). The expression levels of B cells naive, B cells memory, T cells CD8, CD4 memory resting T cells, M0 macrophages, and eosinophils were significantly different between the 37 cluster-related differentially expressed gene subgroups (**Figure 9B**). There were significant differences in the expression levels of the stromal score, CD4 memory resting T cells, follicular helper T cells, activated NK cells, monocytes, M0 macrophages, M1 macrophages, resting mast cells, eosinophils, and neutrophils in high- and low-risk groups (**Figure 9C**). The risk score was significantly correlated with the levels of the stromal score, CD4 memory resting T cells, CD4 memory activated T cells, follicular helper T cells, M0 macrophages, M1 macrophages, and resting mast cells (**Figures 9D–I** and **Figure S6**).

**Figure 10:**
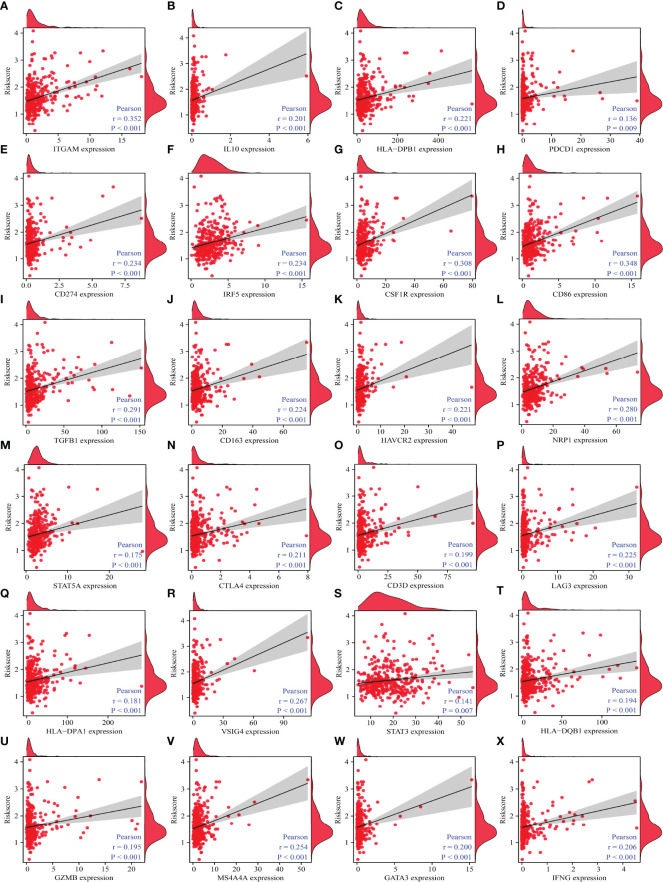
The correlation between the risk model and HCC immune-infiltrating cell markers. **(A)** ITGAM; **(B)** IL10; **(C)** HLA-DPB1; **(D)** PDCD1; **(E)** CD274; **(F)** IRF5; **(G)**; CSF1R; **(H)** CD86; **(I)** TGFB1; **(J)** CD163; **(K)** HAVCR2; **(L)** NRP1; **(M)** STAT5A; **(N)** CTLA4; **(O)** CD3D; **(P)** LAG3; **(Q)** HLA-DPA1; **(R)** VSIG4; **(S)** STAT3; **(T)** HLA-DQB1; **(U)** GZMB; **(V)** MS4A4A; **(W)** GATA3; **(X)** IFNG. HCC, hepatocellular carcinoma.

**Figure 11:**
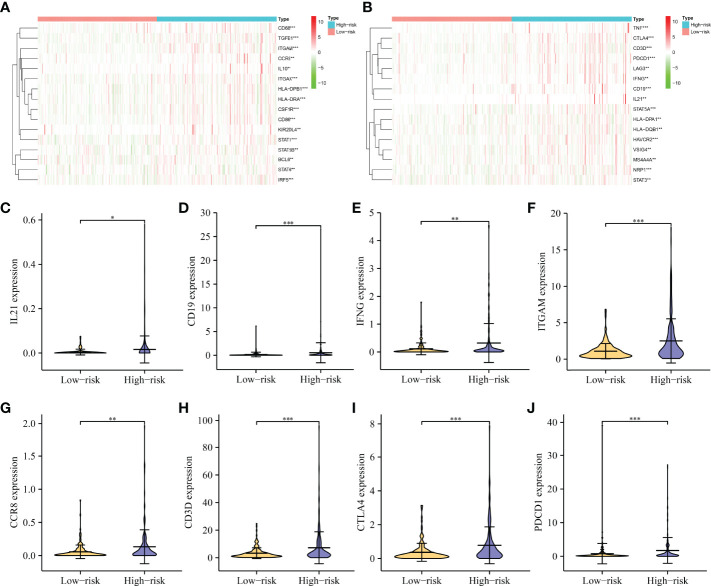
The expression levels of immune-infiltrating cell markers in high- and low-risk groups. **(A, B)** A heatmap showing the expression pattern of immune genes in HCC tissues. **(C–J)** Violin showing the expression of IL21, CD19, IFNG, ITGAM, CCR8, CD3D, CTLA4, and PDCD1 in HCC tissues. HCC, hepatocellular carcinoma; *P < 0.05; **P < 0.01; ***P < 0.001.

Correlation analysis revealed that immune cell markers IL10, ITGAM, STAT5B, CD68, HLA-DPB1, KIR2DL4, IRF5, CSF1R, CD274, HLA-DRA, CD8B, STAT1, NOS2, ITGAX, CD86, CD8A, BCL6, TGFB1, CD163, CCR8, TBX21, CCL2, CD3E, TNF, CD1C, CD2, HAVCR2, NRP1, STAT5A, CD3D, LAG3, HLA-DPA1, PDCD1, VSIG4, STAT3, GZMB, MS4A4A, GATA3, IFNG, and HLA-DQB1 expression levels were associated with the risk score level in HCC (**Figures 10** and **S7**), and the expression levels of IRF5, ITGAM, CD86, NRP1, CTLA4, STAT5A, HAVCR2, CSF1R, STAT1, CD19, ITGAX, CD68, TGFB1, CD3D, PDCD1, HLA-DPB1, HLA-DRA, TNF, CCR8, IFNG, VSIG4, BCL6, IL10, HLA-DPA1, STAT5B, MS4A4A, STAT6, LAG3, HLA-DQB1, KIR2DL4, STAT3, and IL21 differed significantly between the high- and low-risk groups (**Figure 11**).

## Discussion

Numerous studies indicate that the expression levels of many genes change during HCC progression (23–25). Wang et al. for example discovered that the expression level of MAGEH1 is significantly downregulated in HCC tissues, which is associated with poor prognosis in HCC patients. MAGEH1 can inhibit proliferation, migration, and invasion and delay HCC progression (24). Yang et al. reported that elevated SHOX2 expression is associated with tumor recurrence and TNM stage in HCC patients. Increased SHOX2 expression is observed in HCC cells. SHOX2 expression can be suppressed to inhibit HCC cell proliferation and invasion (25).

Current research demonstrates that RNA modification is associated with the progression of malignant tumors (12–16, 26, 27). For example, the level of IGF2BP3 overexpression correlates with cancer progression and survival. IGF2BP3 knockdown inhibits DNA replication in the S phase of the cell cycle, cell proliferation, and angiogenesis by reading the m6A modification of CCND1 and VEGF (12). WTAP is highly expressed and is a risk factor for poor prognosis in HCC patients. WTAP can promote cell proliferation and tumor growth in HCC patients. ETS1 is a downstream effector of WTAP. WTAP can induce post-transcriptional repression of ETS1 by regulating m6A modification, which in turn mediates the p21/p27-dependent mechanism involved in the G2/M phase regulation of HCC cells (26). The expression level of METTL3 is significantly elevated in HCC tissues and cells. Elevated METTL3 expression is associated with poor OS. When METTL3 expression is inhibited, the ability of HCC cells to invade, migrate, and proliferate is significantly decreased. Mechanistically, METTL3 may regulate the expression level of USP7 *via* m6A methylation modification, thereby promoting the growth and migration of HCC cells (27). This suggests that m6A/m1A/m5C regulated genes play important roles in HCC progression. In this study, the roles of 45 m6A/m1A/m5C regulated genes were explored in HCC progression, and significant differences in OS, clinical stage, T stage, tumor grade, and fustat status between subgroups of m6A/m1A/m5C regulated genes were observed. The identification of the expression of the risk model genes YBX1, ZC3H13, YTHDF1, TRMT10C, YTHDF2, RRP8, TRMT6, LRPPRC, and IGF2BP3 in HCC tissues was consistent with that in TCGA database. In addition, a high-risk score constructed using YBX1, ZC3H13, YTHDF1, TRMT10C, YTHDF2, RRP8, TRMT6, LRPPRC, and IGF2BP3 was associated with poor prognosis, tumor grade, clinical stage, T stage, distant metastasis, and fustat status and is an independent risk factor for poor prognosis in HCC patients. Preliminary evidence suggests that m6A/m1A/m5C regulated genes play important biological roles in the progression of HCC, which can be used to determine the prognosis and survival of HCC patients.

The signaling pathways, including the cell cycle, DNA replication, and WNT are closely associated with cancer progression (28–34). Previous studies have found that ASF1B is highly expressed in HCC, and elevated ASF1B expression level is associated with poor prognosis in HCC patients. GSEA revealed that ASF1B may regulate the cell cycle, DNA replication, and oocyte meiotic signaling pathway. ASF1B silencing inhibits the growth and cell cycle arrest, induces apoptosis, and reduces the expression levels of PCNA, cyclinB1, cyclinE2, and CDK9 in HCC cells (28). The level of TCF3 expression is significantly increased in HCC tissues. TCF3 expression level correlates with clinical stage, tumor size, TNM stage, grade, OS, disease-specific survival, and progression-free survival (PFS) in HCC patients. TCF3 knockdown inhibits cancer cell proliferation and cell cycle, which is associated with dysregulation of the WNT signaling mechanism (31). m6A/m1A/m5C regulated genes are involved in multiple functions, including RNA modification and methylation, mRNA methylation and modification, regulation of RNA, and mRNA stability. The cell cycle, base excision repair, RNA degradation, oocyte meiosis, DNA replication, homologous recombination, basal transcription factors, pathways in cancer, WNT signaling pathway, and long-term potentiation processes are involved in the construction of a high-risk model for m6A/m1A/m5C regulated genes. Previous studies indicate that m6A/m1A/m5C regulated genes are closely associated with HCC progression. However, the mechanism of m6A/m1A/m5C regulated genes in HCC progression remains to be confirmed in future studies.

Currently, targeted therapy and immunotherapy are among the treatment options available to HCC patients (2, 35–38). For example, PD-1 inhibitors are well tolerated by HCC patients. HCC patients have achieved good clinical outcomes. The median OS for PD-1 inhibitor-treated HCC patients is 6.6 months, the median PFS is 5.3 months, and the overall response rate is 30.8%. A patient achieves complete remission (38). The relationship between immunotherapy and the immune microenvironment is well established (39, 40). In this study, the expression levels of CD4 memory resting T cells, follicular helper T cells, activated NK cells, monocytes, M0 macrophages, M1 macrophages, resting mast cells, eosinophils, and neutrophils were found to be significantly different in the risk model constructed by m6A/m1A/m5C regulated genes YBX1, ZC3H13, YTHDF1, TRMT10C, YTHDF2, RRP8, TRMT6, LRPPRC, and IGF2BP3. The risk score correlated with levels of the stromal score, CD4 memory resting T cells, CD4 memory activated T cells, follicular helper T cells, M0 macrophages, M1 macrophages, and resting mast cells. In addition, the risks core correlated with the HCC immune cell markers, including IL10, ITGAM, STAT5B, CD68, HLA-DPB1, KIR2DL4, IRF5, CSF1R, CD274, HLA-DRA, CD8B, STAT1, NOS2, ITGAX, CD86, CD8A, BCL6, TGFB1, CD163, CCR8, TBX21, CCL2, CD3E, TNF, CD1C, CD2, HAVCR2, NRP1, STAT5A, CD3D, LAG3, HLA-DPA1, PDCD1, VSIG4, STAT3, GZMB, MS4A4A, GATA3, IFNG, and HLA-DQB1. This suggests that the risk model constructed using m6A/m1A/m5C regulated genes is associated with HCC immune cell infiltration.

This study identified the involvement of m6A/m5C/m1A regulated genes and constructed a risk model for HCC using HCC data from TCGA database and our hospital data. In the future, however, this must be confirmed using additional clinical HCC tissue samples and cell experiments. m6A/m5C/m1A regulated genes are generally involved in the occurrence and development of HCC. m6A/m5C/m1A regulated genes YBX1, ZC3H13, YTHDF1, TRMT10C, YTHDF2, RRP8, TRMT6, LRPPRC, and IGF2BP3 are the influencing factors of poor prognosis in HCC patients. A high-risk score is associated with patient prognosis and is an independent risk factor for poor prognosis in HCC patients. The risk score is associated with HCC stromal score and levels of CD4 memory resting T cells, M0 macrophages, M1 macrophages, resting mast cells, CD4 memory activated T cells, and follicular helper T cells.

## Data Availability Statement

The data used in database are available from the TCGA website and the data of qRT-PCR by contacting the corresponding authors.

## Ethics Statement

The studies involving human participants were reviewed and approved by the ethics committee of Affiliated Hospital of Zunyi Medical University. The patients/participants provided their written informed consent to participate in this study. Written informed consent was obtained from the individual(s) for the publication of any potentially identifiable images or data included in this article.

## Author Contributions

DK, R-SS, and DL designed the study and supervised the manuscript writing process. DL, KL, WZ, and K-WY analyzed the data and wrote the manuscript. DL, D-AM, and G-JJ performed data visualization and edited the language of the manuscript. All authors approve the submission and publication of the manuscript.

## Funding

Our research was funded by the Zunyi City Joint Fund (Zun Shi Ke He HZ Word (2021) No. 73).

## Conflict of Interest

The authors declare that the research was conducted in the absence of any commercial or financial relationships that could be construed as a potential conflict of interest.

## Publisher’s Note

All claims expressed in this article are solely those of the authors and do not necessarily represent those of their affiliated organizations, or those of the publisher, the editors and the reviewers. Any product that may be evaluated in this article, or claim that may be made by its manufacturer, is not guaranteed or endorsed by the publisher.

## Abbreviations

GSEA, Gene Set Enrichment Analysis; DEGs, differentially expressed genes; TCGA, The Cancer Genome Atlas; FDR, false discovery rate; HCC, hepatocellular carcinoma; PPI, protein–protein interaction; SORA, sorafenib; m6A, *N* ^6^-methyladenosine; m1A, *N* ^1^-methyladenosine; m5C, 5-methylcytosine; EEOC, endometrioid epithelial ovarian cancer.
