# Supplementary material for: The m6A/m5C/m1A Regulated Gene Signature Predicts the Prognosis and Correlates With the Immune Status of Hepatocellular Carcinoma
Source: Front Immunol. 2022 Jun 27;13:918140. doi: 10.3389/fimmu.2022.918140 (PMC9272990; doi:10.3389/fimmu.2022.918140)
Supplement: Supplementary file 1 [file DataSheet_1.docx]

**Figure S**


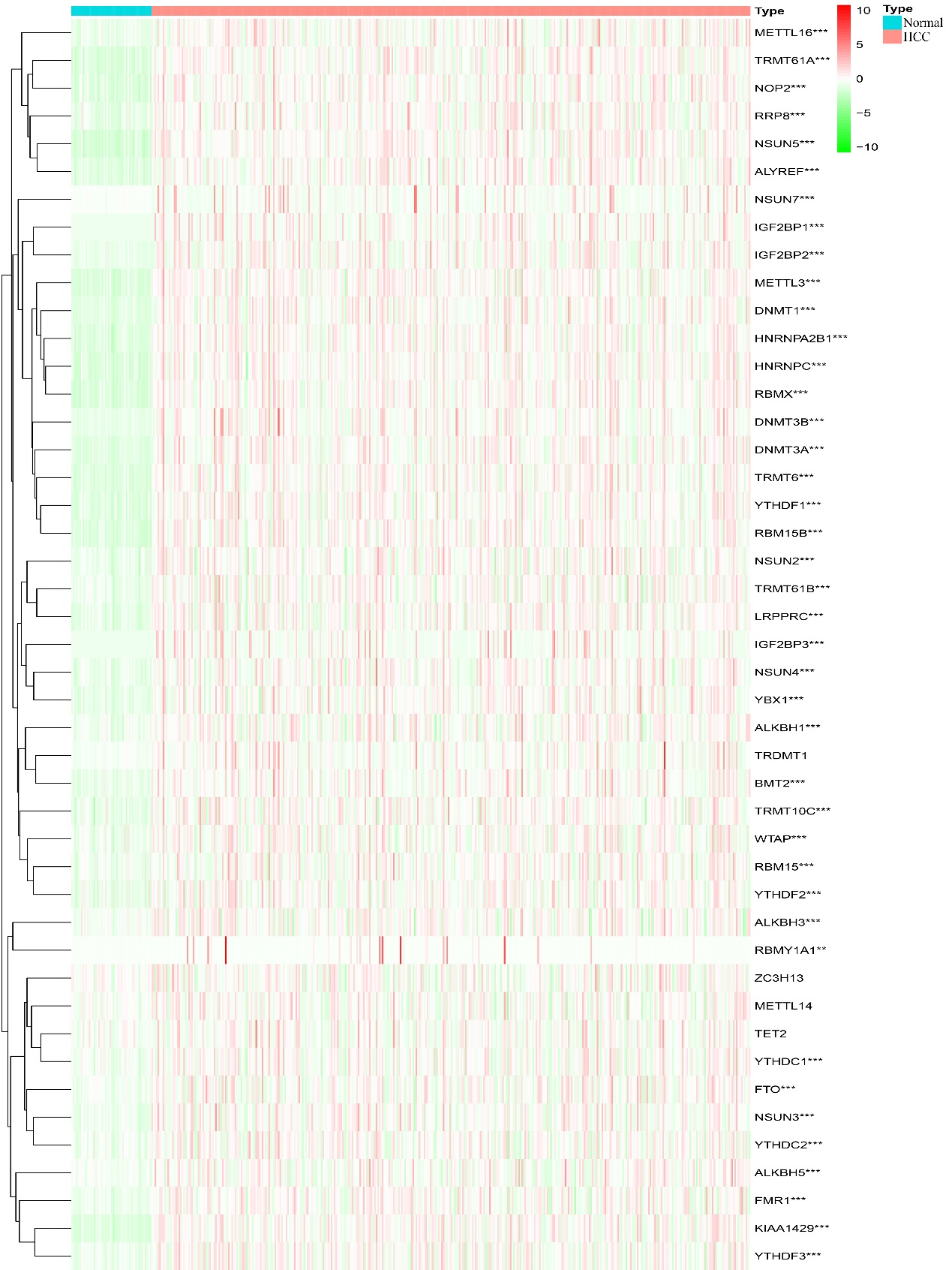


Figure S1. The expression levels of m6A/m1A/m5C regulated genes in HCC tissues.

Note: HCC, hepatocellular carcinoma.


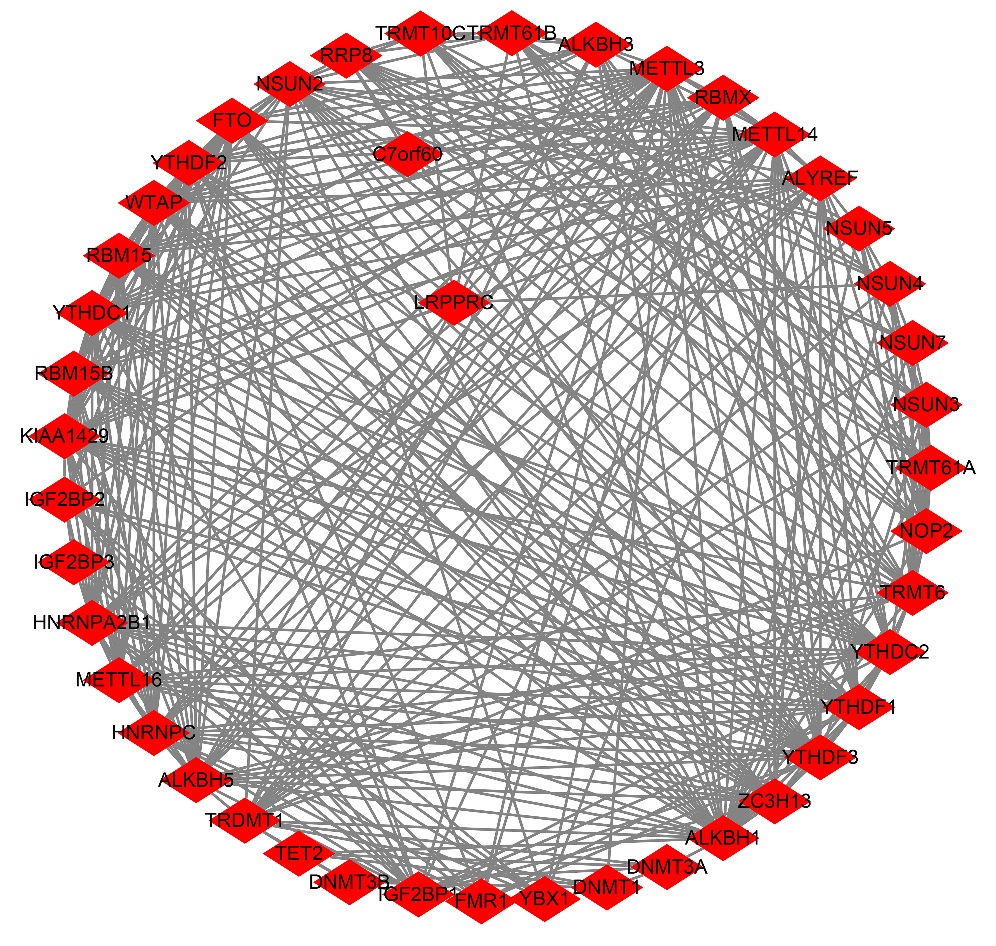


Figure S2. The PPI network of m6A/m1A/m5C regulated genes.

Note: PPI, protein-protein interaction.


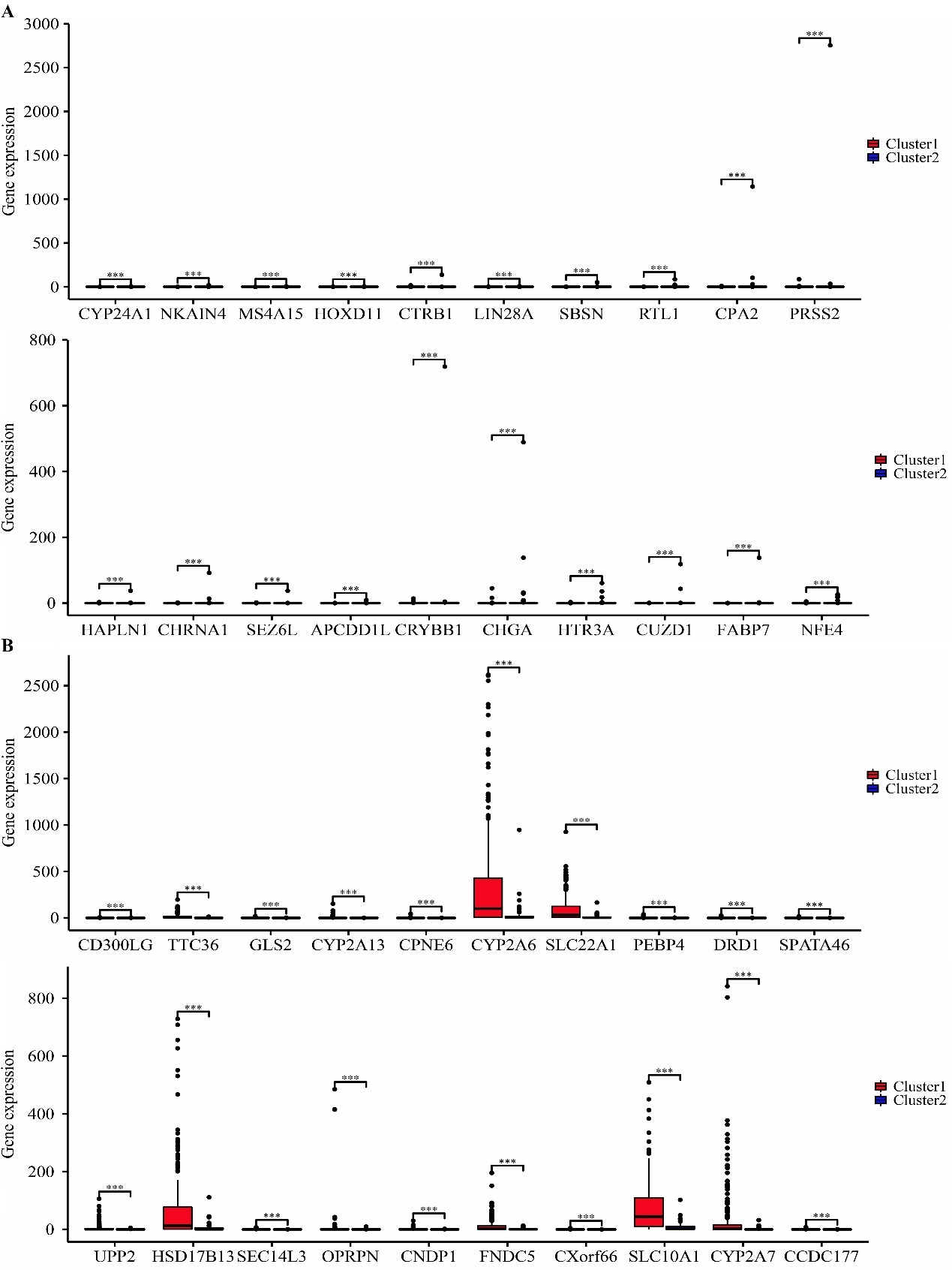


Figure S3. The cluster-related DEGs are showed in HCC tissues. (A) Up-regulated genes; (B) Down-regulated genes.

Note: HCC, hepatocellular carcinoma.


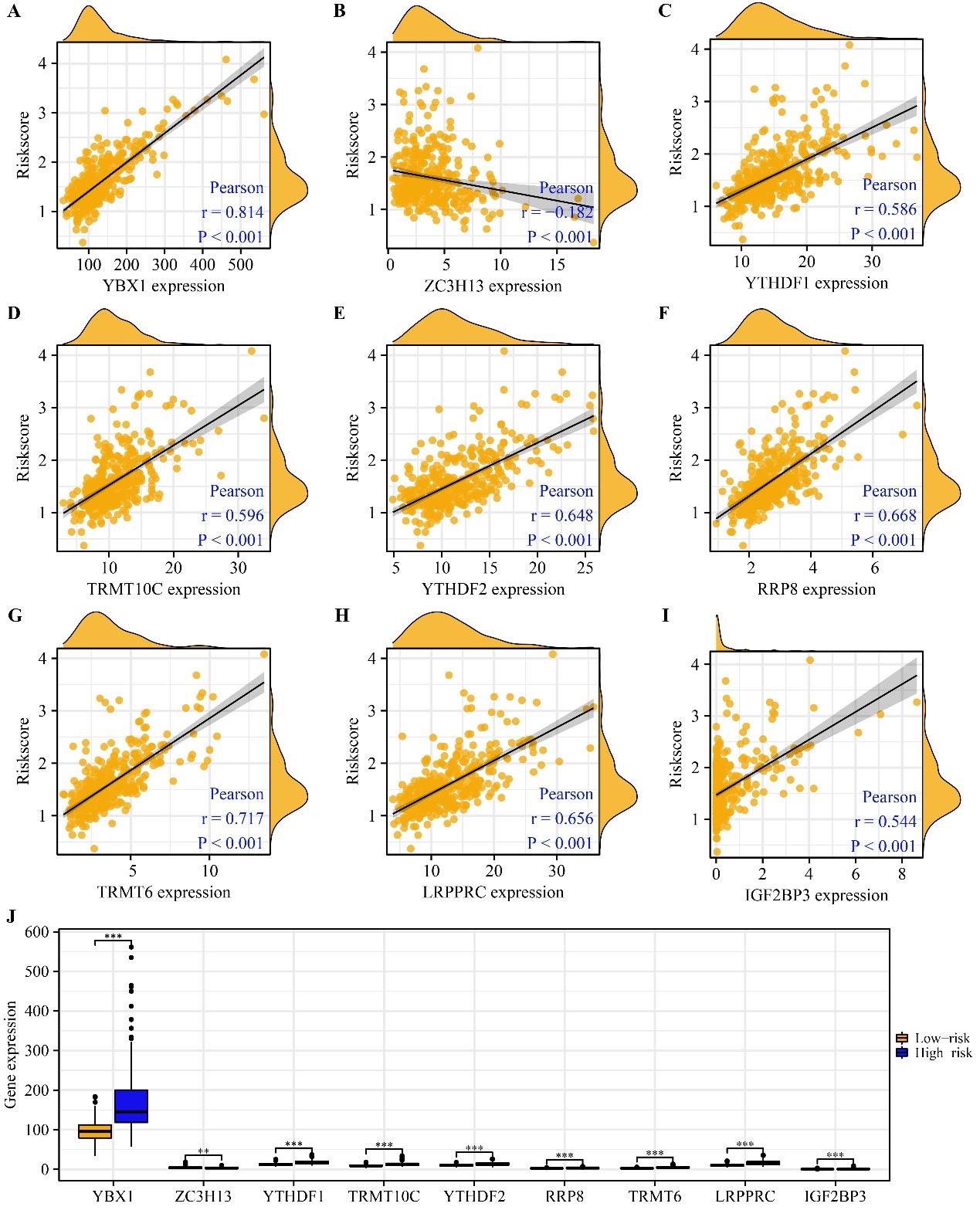


Figure S4. The correlation between the risk score and expression levels of m6A/m1A/m5C regulated genes.


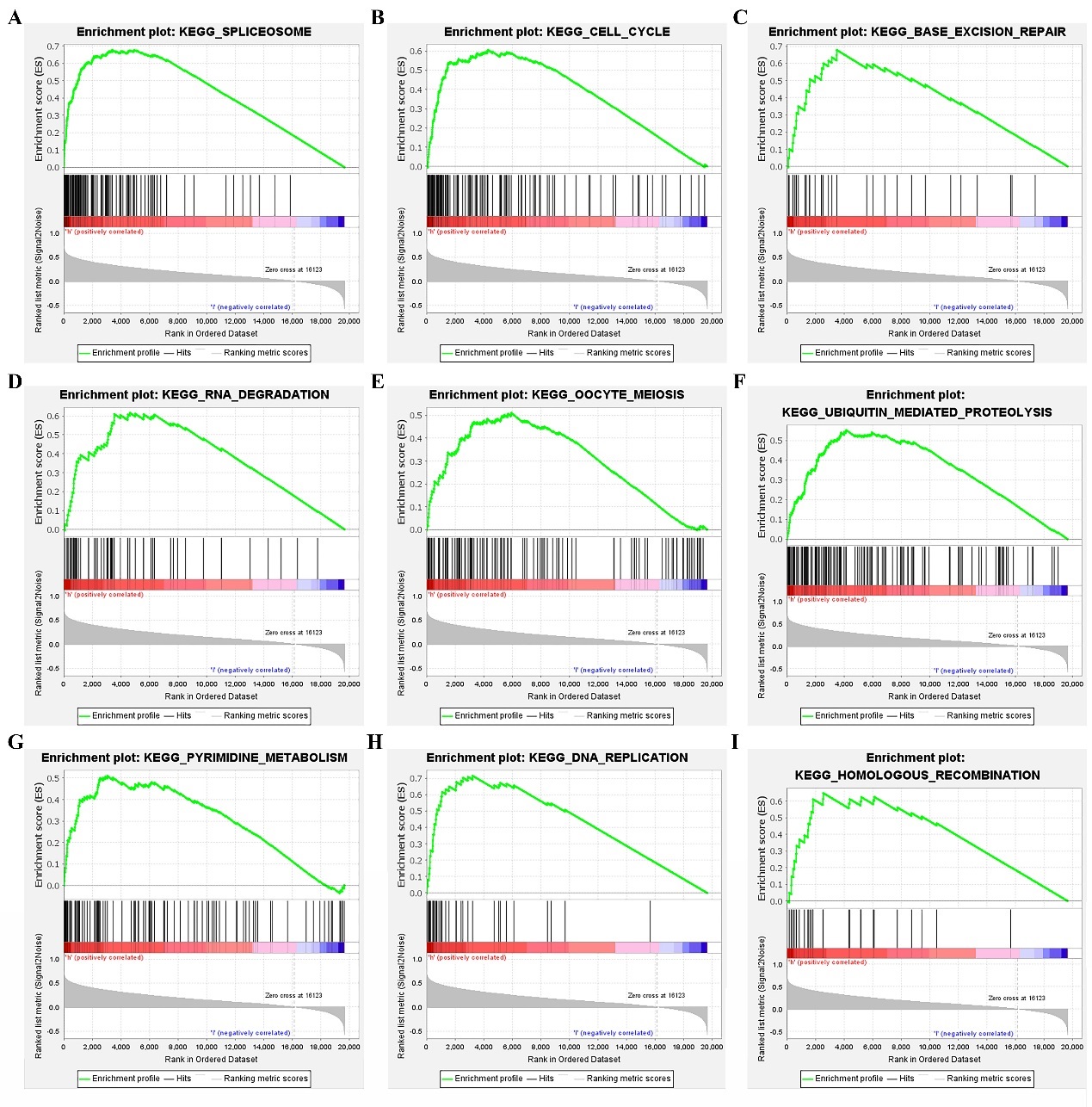


Figure S5. Signaling mechanisms involved in high-riskscore. (A) Spliceosome; (B) Cell cycle; (C) Base excision repair; (D) RNA degradation; (E) Oocyte meiosis; (F) Ubiquitin mediated proteolysis; (G) Pyrimidine metabolism; (H) DNA replication; (I) Homologous recombination.


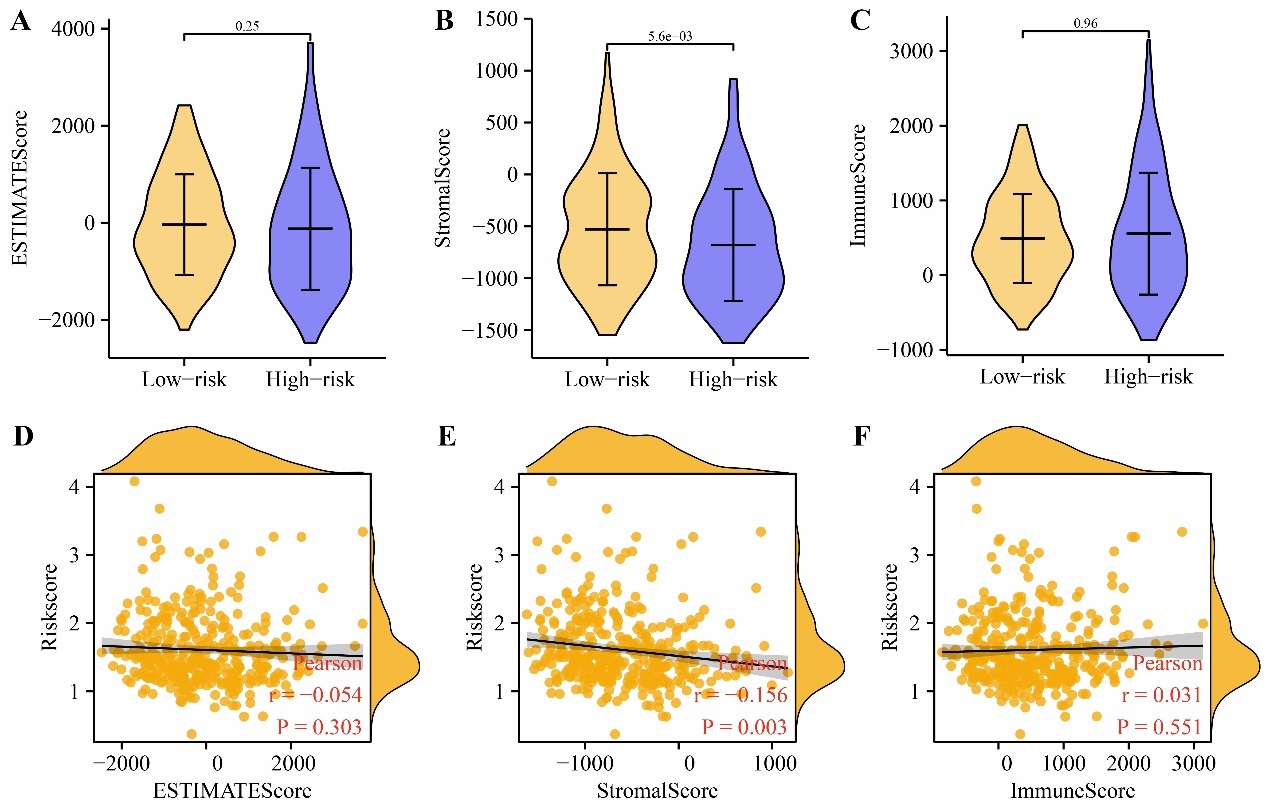


Figure S6. The correlation between the risk score and stromal score in HCC tissues.

Note: HCC, hepatocellular carcinoma.


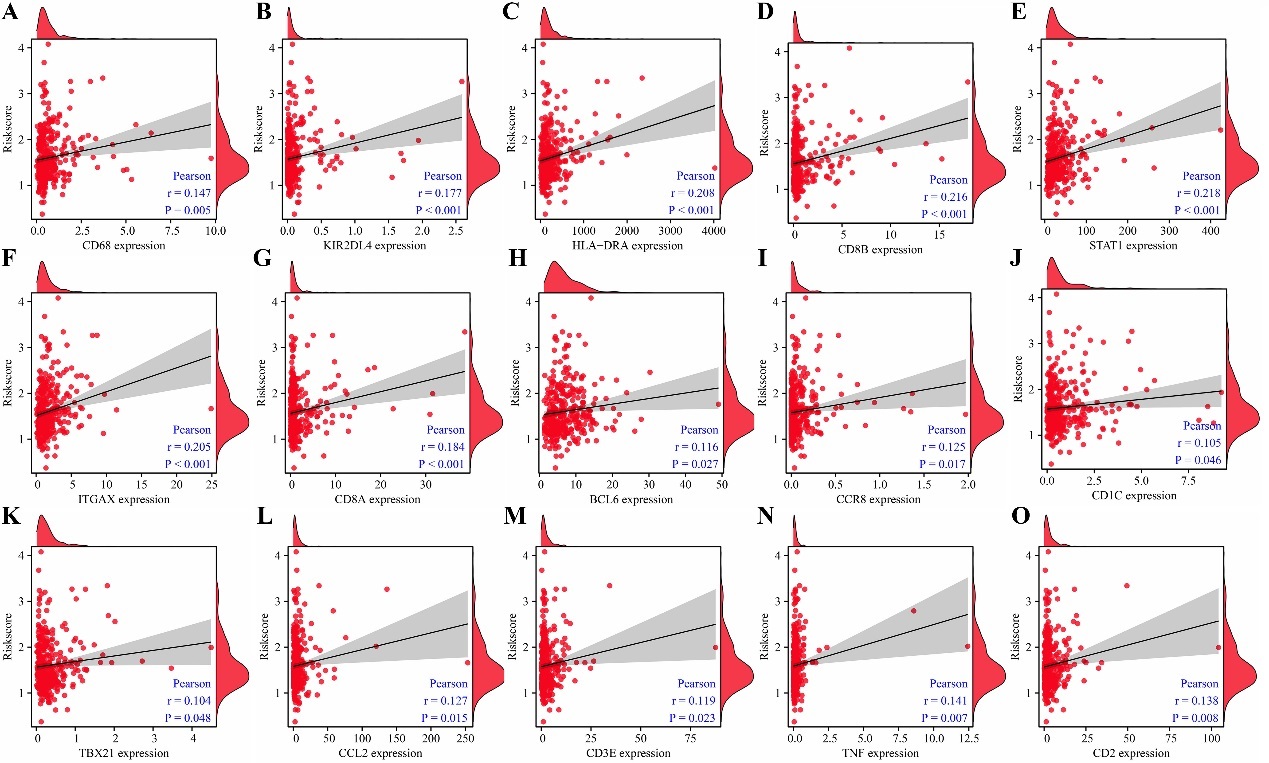


Figure S7. Association of the risk model with HCC immune-infiltrating cell markers. (A) CD68; (B) KIR2DL4; (C) HLA-DRA; (D) CD8B; (E) STAT1; (F) ITGAX; (G) CD8A; (H) BCL6; (I) CCR8; (J) CD1C; (K) TBX21; (L) CCL2; (M) CD3E; (N) TNF; (O) CD2.

Note: HCC, hepatocellular carcinoma.

**Table** **S**

Table S1. The functions of m6A/m1A/m5C regulated genes.

| Ontology | ID | Description |
| --- | --- | --- |
| BP | GO:0009451 | RNA modification |
| BP | GO:0001510 | RNA methylation |
| BP | GO:0032259 | methylation |
| BP | GO:0043414 | macromolecule methylation |
| BP | GO:1903311 | regulation of mRNA metabolic process |
| BP | GO:0080009 | mRNA methylation |
| BP | GO:0016556 | mRNA modification |
| BP | GO:0043488 | regulation of mRNA stability |
| BP | GO:0034470 | ncRNA processing |
| BP | GO:0043487 | regulation of RNA stability |
| BP | GO:0061013 | regulation of mRNA catabolic process |
| BP | GO:0006417 | regulation of translation |
| BP | GO:0034248 | regulation of cellular amide metabolic process |
| BP | GO:0008380 | RNA splicing |
| BP | GO:0000377 | RNA splicing, via transesterification reactions with bulged adenosine as nucleophile |
| BP | GO:0000398 | mRNA splicing, via spliceosome |
| BP | GO:0000375 | RNA splicing, via transesterification reactions |
| BP | GO:0006401 | RNA catabolic process |
| BP | GO:0031167 | rRNA methylation |
| BP | GO:0048024 | regulation of mRNA splicing, via spliceosome |
| BP | GO:0006402 | mRNA catabolic process |
| BP | GO:0051028 | mRNA transport |
| BP | GO:0000154 | rRNA modification |
| BP | GO:0030488 | tRNA methylation |
| BP | GO:0050657 | nucleic acid transport |
| BP | GO:0050658 | RNA transport |
| BP | GO:0006400 | tRNA modification |
| BP | GO:0051236 | establishment of RNA localization |
| BP | GO:0043484 | regulation of RNA splicing |
| BP | GO:0050684 | regulation of mRNA processing |
| BP | GO:0017148 | negative regulation of translation |
| BP | GO:0061157 | mRNA destabilization |
| BP | GO:0050779 | RNA destabilization |
| BP | GO:0034249 | negative regulation of cellular amide metabolic process |
| BP | GO:0006403 | RNA localization |
| BP | GO:0015931 | nucleobase-containing compound transport |
| BP | GO:0006304 | DNA modification |
| BP | GO:1903313 | positive regulation of mRNA metabolic process |
| BP | GO:0008033 | tRNA processing |
| BP | GO:0070989 | oxidative demethylation |
| BP | GO:0061014 | positive regulation of mRNA catabolic process |
| BP | GO:0044728 | DNA methylation or demethylation |
| BP | GO:0070129 | regulation of mitochondrial translation |
| BP | GO:0006307 | DNA dealkylation involved in DNA repair |
| BP | GO:0000381 | regulation of alternative mRNA splicing, via spliceosome |
| BP | GO:0035510 | DNA dealkylation |
| BP | GO:0006399 | tRNA metabolic process |
| BP | GO:0045727 | positive regulation of translation |
| BP | GO:0000380 | alternative mRNA splicing, via spliceosome |
| BP | GO:0006446 | regulation of translational initiation |
| BP | GO:1903312 | negative regulation of mRNA metabolic process |
| BP | GO:0006364 | rRNA processing |
| BP | GO:0034250 | positive regulation of cellular amide metabolic process |
| BP | GO:0009048 | dosage compensation by inactivation of X chromosome |
| BP | GO:0016072 | rRNA metabolic process |
| BP | GO:0007549 | dosage compensation |
| BP | GO:1902369 | negative regulation of RNA catabolic process |
| BP | GO:0080111 | DNA demethylation |
| BP | GO:0042254 | ribosome biogenesis |
| BP | GO:0070988 | demethylation |
| BP | GO:0045948 | positive regulation of translational initiation |
| BP | GO:0070475 | rRNA base methylation |
| BP | GO:0140053 | mitochondrial gene expression |
| BP | GO:0060339 | negative regulation of type I interferon-mediated signaling pathway |
| BP | GO:0006413 | translational initiation |
| BP | GO:0006406 | mRNA export from nucleus |
| BP | GO:0071427 | mRNA-containing ribonucleoprotein complex export from nucleus |
| BP | GO:1902373 | negative regulation of mRNA catabolic process |
| BP | GO:0071426 | ribonucleoprotein complex export from nucleus |
| BP | GO:0071166 | ribonucleoprotein complex localization |
| BP | GO:0006405 | RNA export from nucleus |
| BP | GO:0032543 | mitochondrial translation |
| BP | GO:0031331 | positive regulation of cellular catabolic process |
| BP | GO:2000765 | regulation of cytoplasmic translation |
| BP | GO:0009896 | positive regulation of catabolic process |
| BP | GO:0006611 | protein export from nucleus |
| BP | GO:0060338 | regulation of type I interferon-mediated signaling pathway |
| BP | GO:0051168 | nuclear export |
| BP | GO:0000959 | mitochondrial RNA metabolic process |
| BP | GO:0048255 | mRNA stabilization |
| BP | GO:0051445 | regulation of meiotic cell cycle |
| BP | GO:0043489 | RNA stabilization |
| BP | GO:0045814 | negative regulation of gene expression, epigenetic |
| BP | GO:0051573 | negative regulation of histone H3-K9 methylation |
| BP | GO:2000767 | positive regulation of cytoplasmic translation |
| BP | GO:0051568 | histone H3-K4 methylation |
| BP | GO:0060019 | radial glial cell differentiation |
| BP | GO:0045824 | negative regulation of innate immune response |
| BP | GO:0031330 | negative regulation of cellular catabolic process |
| BP | GO:0090646 | mitochondrial tRNA processing |
| BP | GO:0031503 | protein-containing complex localization |
| BP | GO:0019827 | stem cell population maintenance |
| BP | GO:0098727 | maintenance of cell number |
| BP | GO:0044827 | modulation by host of viral genome replication |
| BP | GO:0051571 | positive regulation of histone H3-K4 methylation |
| BP | GO:0006305 | DNA alkylation |
| BP | GO:0006306 | DNA methylation |
| BP | GO:0000963 | mitochondrial RNA processing |
| BP | GO:0070131 | positive regulation of mitochondrial translation |
| BP | GO:0070525 | tRNA threonylcarbamoyladenosine metabolic process |
| BP | GO:0001960 | negative regulation of cytokine-mediated signaling pathway |
| BP | GO:2001252 | positive regulation of chromosome organization |
| BP | GO:0009895 | negative regulation of catabolic process |
| BP | GO:0033189 | response to vitamin A |
| BP | GO:0048025 | negative regulation of mRNA splicing, via spliceosome |
| BP | GO:0060761 | negative regulation of response to cytokine stimulus |
| BP | GO:0031061 | negative regulation of histone methylation |
| BP | GO:0007219 | Notch signaling pathway |
| BP | GO:0033044 | regulation of chromosome organization |
| BP | GO:0002097 | tRNA wobble base modification |
| BP | GO:0006913 | nucleocytoplasmic transport |
| BP | GO:0031058 | positive regulation of histone modification |
| BP | GO:0051169 | nuclear transport |
| BP | GO:0051570 | regulation of histone H3-K9 methylation |
| BP | GO:0033119 | negative regulation of RNA splicing |
| BP | GO:0060337 | type I interferon signaling pathway |
| BP | GO:0071357 | cellular response to type I interferon |
| BP | GO:0034340 | response to type I interferon |
| BP | GO:0002181 | cytoplasmic translation |
| BP | GO:0006346 | methylation-dependent chromatin silencing |
| BP | GO:1905269 | positive regulation of chromatin organization |
| BP | GO:0051569 | regulation of histone H3-K4 methylation |
| BP | GO:0050686 | negative regulation of mRNA processing |
| BP | GO:0044788 | modulation by host of viral process |
| BP | GO:0034968 | histone lysine methylation |
| BP | GO:0042035 | regulation of cytokine biosynthetic process |
| BP | GO:0051321 | meiotic cell cycle |
| BP | GO:0006342 | chromatin silencing |
| BP | GO:0002183 | cytoplasmic translational initiation |
| BP | GO:0042089 | cytokine biosynthetic process |
| BP | GO:0042107 | cytokine metabolic process |
| BP | GO:0051567 | histone H3-K9 methylation |
| BP | GO:0018022 | peptidyl-lysine methylation |
| BP | GO:0031062 | positive regulation of histone methylation |
| BP | GO:0022618 | ribonucleoprotein complex assembly |
| BP | GO:0016571 | histone methylation |
| BP | GO:0031057 | negative regulation of histone modification |
| BP | GO:0045746 | negative regulation of Notch signaling pathway |
| BP | GO:0031056 | regulation of histone modification |
| BP | GO:0071826 | ribonucleoprotein complex subunit organization |
| BP | GO:2000241 | regulation of reproductive process |
| BP | GO:2001251 | negative regulation of chromosome organization |
| BP | GO:0016569 | covalent chromatin modification |
| BP | GO:0035196 | production of miRNAs involved in gene silencing by miRNA |
| BP | GO:0048599 | oocyte development |
| BP | GO:0050777 | negative regulation of immune response |
| BP | GO:0061647 | histone H3-K9 modification |
| BP | GO:0009994 | oocyte differentiation |
| BP | GO:0031050 | dsRNA processing |
| BP | GO:0070918 | production of small RNA involved in gene silencing by RNA |
| BP | GO:0006376 | mRNA splice site selection |
| BP | GO:0042220 | response to cocaine |
| BP | GO:0001959 | regulation of cytokine-mediated signaling pathway |
| BP | GO:0006479 | protein methylation |
| BP | GO:0008213 | protein alkylation |
| BP | GO:1903902 | positive regulation of viral life cycle |
| BP | GO:1904356 | regulation of telomere maintenance via telomere lengthening |
| BP | GO:1905268 | negative regulation of chromatin organization |
| BP | GO:1902275 | regulation of chromatin organization |
| BP | GO:0042255 | ribosome assembly |
| BP | GO:0022412 | cellular process involved in reproduction in multicellular organism |
| BP | GO:0060759 | regulation of response to cytokine stimulus |
| BP | GO:0031060 | regulation of histone methylation |
| BP | GO:0021872 | forebrain generation of neurons |
| BP | GO:0071230 | cellular response to amino acid stimulus |
| CC | GO:0034708 | methyltransferase complex |
| CC | GO:0016607 | nuclear speck |
| CC | GO:0035770 | ribonucleoprotein granule |
| CC | GO:0071013 | catalytic step 2 spliceosome |
| CC | GO:0005681 | spliceosomal complex |
| CC | GO:0000791 | euchromatin |
| CC | GO:0036464 | cytoplasmic ribonucleoprotein granule |
| CC | GO:0010494 | cytoplasmic stress granule |
| CC | GO:0000792 | heterochromatin |
| CC | GO:0098687 | chromosomal region |
| CC | GO:0005719 | nuclear euchromatin |
| CC | GO:0005635 | nuclear envelope |
| CC | GO:0005759 | mitochondrial matrix |
| CC | GO:0031965 | nuclear membrane |
| CC | GO:0009295 | nucleoid |
| CC | GO:0042645 | mitochondrial nucleoid |
| CC | GO:0000315 | organellar large ribosomal subunit |
| CC | GO:0005762 | mitochondrial large ribosomal subunit |
| CC | GO:0000775 | chromosome, centromeric region |
| CC | GO:0000790 | nuclear chromatin |
| MF | GO:0008168 | methyltransferase activity |
| MF | GO:0016741 | transferase activity, transferring one-carbon groups |
| MF | GO:0008757 | S-adenosylmethionine-dependent methyltransferase activity |
| MF | GO:0008173 | RNA methyltransferase activity |
| MF | GO:0140098 | catalytic activity, acting on RNA |
| MF | GO:0008174 | mRNA methyltransferase activity |
| MF | GO:0008175 | tRNA methyltransferase activity |
| MF | GO:0140101 | catalytic activity, acting on a tRNA |
| MF | GO:0008649 | rRNA methyltransferase activity |
| MF | GO:0140102 | catalytic activity, acting on a rRNA |
| MF | GO:0016706 | oxidoreductase activity, acting on paired donors, with incorporation or reduction of molecular oxygen, 2-oxoglutarate as one donor, and incorporation of one atom each of oxygen into both donors |
| MF | GO:0003730 | mRNA 3'-UTR binding |
| MF | GO:0008198 | ferrous iron binding |
| MF | GO:0048027 | mRNA 5'-UTR binding |
| MF | GO:0032451 | demethylase activity |
| MF | GO:0051213 | dioxygenase activity |
| MF | GO:0140097 | catalytic activity, acting on DNA |
| MF | GO:0000049 | tRNA binding |
| MF | GO:0045182 | translation regulator activity |
| MF | GO:0016705 | oxidoreductase activity, acting on paired donors, with incorporation or reduction of molecular oxygen |
| MF | GO:0043022 | ribosome binding |
| MF | GO:0005506 | iron ion binding |
| MF | GO:0035613 | RNA stem-loop binding |
| MF | GO:0003727 | single-stranded RNA binding |
| MF | GO:0008266 | poly(U) RNA binding |
| MF | GO:0035198 | miRNA binding |
| MF | GO:0003697 | single-stranded DNA binding |
| MF | GO:0008187 | poly-pyrimidine tract binding |
| MF | GO:0061980 | regulatory RNA binding |
| MF | GO:0043021 | ribonucleoprotein complex binding |
| MF | GO:0035064 | methylated histone binding |
| MF | GO:0140034 | methylation-dependent protein binding |

Note: BP, biological process; CC, cellular component; MF, molecular function; DEGs, differentially expressed genes.

Table S2. The cluster-related DEGs in HCC tissues.

| Gene | LogFC | FDR |
| --- | --- | --- |
| GJA3 | 3.116067379 | 3.44E-08 |
| CD300LG | -2.999646843 | 2.44E-09 |
| CYP7A1 | -2.127466909 | 3.63E-08 |
| ZBBX | 2.674603622 | 1.76E-09 |
| TRPA1 | 2.899745029 | 0.000507927 |
| MAPK15 | 2.741791195 | 2.88E-05 |
| CYP24A1 | 4.446614723 | 5.65E-07 |
| BOLL | 2.032154258 | 2.52E-06 |
| DRP2 | 2.461555598 | 2.32E-08 |
| FIBCD1 | 2.086441896 | 0.000682017 |
| G6PD | 2.433186534 | 1.51E-19 |
| KLRC2 | 2.209204917 | 9.46E-06 |
| TTC36 | -3.220303799 | 4.29E-09 |
| GPRC6A | 2.785676219 | 4.89E-08 |
| INSM1 | 2.855873121 | 0.000820195 |
| NKAIN4 | 4.396286306 | 2.27E-05 |
| CCDC74B | 2.694158673 | 4.70E-06 |
| NEUROG3 | 3.602484702 | 3.86E-05 |
| GLS2 | -3.152791266 | 9.82E-08 |
| NPPB | 2.250989528 | 6.35E-07 |
| PNPLA1 | 2.139432699 | 1.10E-05 |
| GABRA2 | 2.007420035 | 4.76E-06 |
| C7orf57 | 2.033890352 | 0.000271681 |
| FOXF2 | 2.328068809 | 0.000647691 |
| CYP2A13 | -4.77639549 | 3.12E-10 |
| PCK1 | -2.252944438 | 2.04E-11 |
| SLC51A | -2.010403792 | 1.82E-10 |
| MS4A15 | 5.607381482 | 5.05E-08 |
| NCCRP1 | 4.017716273 | 6.55E-06 |
| FAM163A | 2.101279817 | 3.63E-06 |
| HPDL | 2.270408475 | 1.91E-08 |
| FABP6 | 3.518358672 | 2.80E-09 |
| VCX | 2.051539148 | 0.000670023 |
| HOXB8 | 3.356159642 | 0.000165164 |
| HOXD11 | 4.478025696 | 8.57E-06 |
| NTRK1 | 2.98061258 | 0.000212411 |
| DLL3 | 4.194113081 | 7.67E-11 |
| CD177 | 4.348737075 | 6.16E-05 |
| GPM6B | 2.079034561 | 1.68E-05 |
| TRIP13 | 2.065897921 | 3.48E-17 |
| CYP8B1 | -2.629229992 | 4.37E-11 |
| CPNE6 | -4.048300981 | 0.000385198 |
| SEMA3E | 2.494212435 | 2.27E-05 |
| ELFN1 | -2.019483956 | 4.63E-08 |
| PPFIA4 | 2.576080011 | 2.24E-12 |
| NEFM | 2.487379725 | 0.000382401 |
| CYP2A6 | -3.162995879 | 1.87E-10 |
| ANKRD18B | 2.260878142 | 7.99E-06 |
| PANX3 | 2.908452428 | 0.000100157 |
| ANKLE1 | 2.330434497 | 4.60E-08 |
| TRDN | 2.268548243 | 0.000186381 |
| ABCB5 | 2.536751342 | 0.000152401 |
| STAC | 2.789392047 | 7.20E-05 |
| TMSB15A | 3.591123792 | 0.00011345 |
| GPR63 | 2.021624671 | 0.000194655 |
| CDC20 | 2.017759681 | 3.61E-17 |
| BRS3 | 2.37145607 | 0.000196974 |
| SHISA3 | -2.22322703 | 0.00012082 |
| COL8A2 | 2.049010794 | 0.000813036 |
| SLC22A1 | -3.254491636 | 1.57E-11 |
| PGLYRP2 | -2.15480997 | 2.05E-11 |
| TPBG | 2.014774219 | 0.000759639 |
| TRIM54 | 2.091488396 | 3.65E-08 |
| VWDE | 2.178916119 | 0.000367803 |
| SLC38A5 | 2.061672515 | 3.36E-06 |
| GIPR | 2.04123378 | 7.37E-08 |
| PLBD1 | 2.262635316 | 7.27E-10 |
| DCHS2 | 2.180464547 | 5.84E-06 |
| CTRB1 | 4.955363414 | 2.38E-05 |
| SLC2A1 | 2.077322111 | 3.58E-13 |
| PRUNE2 | 2.477868417 | 0.000217361 |
| SCG3 | 3.291785447 | 1.89E-05 |
| PEBP4 | -3.409857343 | 7.10E-06 |
| TEKT2 | 2.853407266 | 8.06E-05 |
| MAGEA4 | 4.371886436 | 1.59E-08 |
| DMRT2 | 2.298390262 | 9.58E-06 |
| ERICH4 | 2.341355892 | 0.00011259 |
| CCNJL | 2.1783089 | 1.20E-05 |
| LIN28A | 4.625228977 | 0.000753055 |
| PTPRN | 2.220969769 | 3.23E-08 |
| KCNK2 | 3.020283638 | 0.000581979 |
| HOXB9 | 2.850316764 | 1.24E-05 |
| PRODH | -2.330360263 | 0.000414684 |
| VWA5B2 | 2.831060122 | 1.73E-06 |
| PITX3 | 2.225890011 | 3.82E-06 |
| PPP1R14C | 2.131903089 | 0.000241763 |
| IBSP | 4.092594739 | 4.61E-06 |
| MYLK2 | 2.134796174 | 5.08E-10 |
| SEPT14 | 3.485373699 | 0.000110731 |
| PKM | 2.221059378 | 2.34E-11 |
| FOXJ1 | 2.179716192 | 1.33E-07 |
| IL31RA | 3.131039334 | 2.14E-05 |
| ANK1 | 2.787449696 | 0.000835057 |
| HEPACAM2 | 3.413288676 | 0.000104232 |
| FGF9 | 2.613601138 | 0.000902273 |
| BIRC7 | 4.155482055 | 2.70E-08 |
| FBN2 | 2.27030732 | 0.000123992 |
| PTHLH | 3.339578016 | 5.03E-06 |
| DRD1 | -2.873376671 | 2.15E-07 |
| HSPA6 | 2.495078958 | 1.97E-06 |
| RNF183 | 2.42240574 | 0.000527581 |
| TLL2 | 2.356982651 | 1.15E-06 |
| UGT8 | 2.455888299 | 3.03E-07 |
| TM4SF19 | 2.388645457 | 3.16E-06 |
| CALHM3 | 2.335937459 | 8.41E-08 |
| TMEM52B | 2.485313533 | 9.04E-07 |
| PITX2 | 3.027091068 | 5.85E-07 |
| SPIB | 3.948899691 | 1.21E-06 |
| CFHR4 | -2.848138692 | 6.64E-15 |
| PFN2 | 2.323038299 | 1.17E-10 |
| MPP2 | 2.005985565 | 5.39E-08 |
| BEND6 | 2.817140464 | 0.000306665 |
| SBSN | 4.509957839 | 5.43E-06 |
| S100A3 | 2.23741021 | 0.000247184 |
| MSC | 2.161209051 | 0.00044085 |
| FRMD5 | 2.801966016 | 4.83E-09 |
| BCAT1 | 2.455295872 | 6.60E-11 |
| LAMP5 | 3.126206563 | 0.000134352 |
| MLF1 | 2.273140751 | 4.00E-08 |
| GPR84 | 2.563194097 | 6.62E-10 |
| SLC7A10 | 2.48554417 | 0.000468131 |
| PIFO | 2.197566444 | 3.14E-10 |
| EYA4 | 2.157111696 | 1.79E-05 |
| KRT17 | 2.910019968 | 4.82E-07 |
| ARX | 2.06452065 | 0.000156478 |
| TWIST1 | 2.573307523 | 0.000130112 |
| EPHA6 | 2.726735655 | 0.000191857 |
| PLAU | 2.129516732 | 0.000116045 |
| DEFB132 | -2.184034111 | 9.30E-06 |
| SPATA46 | -3.218738759 | 5.44E-05 |
| RTL1 | 6.833165927 | 1.13E-05 |
| GFY | 2.005371482 | 3.97E-08 |
| KIAA1549L | 2.285681366 | 5.92E-06 |
| KIF3C | 2.31275151 | 1.03E-09 |
| IGSF11 | 2.914393923 | 1.86E-05 |
| AGR2 | 2.570741239 | 0.000776564 |
| HPCA | 2.380130318 | 2.99E-05 |
| CACNA1G | 2.24940319 | 9.84E-06 |
| PLTP | 2.029723751 | 3.25E-07 |
| PKIB | 2.038981852 | 7.23E-12 |
| NMU | 2.288461474 | 0.000259263 |
| POPDC3 | 3.063593409 | 2.00E-07 |
| SLC22A7 | -2.274097669 | 2.89E-13 |
| DAW1 | 2.266646764 | 6.95E-06 |
| CYP2C8 | -2.202877215 | 4.03E-10 |
| PRR20G | 3.168535898 | 5.81E-07 |
| KLF14 | 3.010385824 | 0.000171334 |
| TNFAIP6 | 2.914663316 | 3.84E-06 |
| RIPPLY2 | 2.995452758 | 3.52E-07 |
| ANO1 | -2.032195045 | 7.51E-11 |
| GYS2 | -2.397836396 | 1.47E-10 |
| SPINK4 | 3.631924093 | 7.14E-07 |
| CXCL5 | 3.559021521 | 1.61E-08 |
| CTAG2 | 2.270202305 | 1.47E-05 |
| GPLD1 | -2.263754452 | 4.74E-11 |
| RFX6 | 3.022741954 | 3.33E-07 |
| CPA2 | 8.173210941 | 2.13E-10 |
| HMGA2 | 2.785541087 | 5.99E-09 |
| STEAP1B | 2.616705951 | 4.43E-12 |
| NLRP6 | -2.589294182 | 5.61E-11 |
| OR8A1 | 2.310984493 | 5.68E-09 |
| DNER | 2.00019762 | 1.49E-05 |
| NR0B1 | 3.734707643 | 2.64E-09 |
| ANKRD7 | 2.86443581 | 1.27E-06 |
| CFAP61 | 2.481345387 | 3.52E-07 |
| TCN1 | 3.538673659 | 5.85E-07 |
| DLX2 | 2.674193277 | 6.73E-07 |
| SPINK13 | 2.710431748 | 1.11E-05 |
| ZNF280A | 3.237644381 | 2.89E-06 |
| POU3F2 | 2.282047944 | 1.44E-08 |
| ABCA12 | 2.569183436 | 1.64E-05 |
| RASGEF1A | 2.078825405 | 1.24E-08 |
| NKPD1 | 2.191268175 | 4.93E-06 |
| CD1A | 3.108427001 | 1.40E-06 |
| UPP2 | -3.185122747 | 0.000101612 |
| SCN5A | 3.02267177 | 1.79E-06 |
| ALX1 | 3.051821922 | 1.93E-10 |
| THRSP | -2.085269252 | 4.66E-05 |
| CALCR | 3.995871025 | 1.57E-06 |
| CHRNA4 | -2.423000263 | 1.57E-05 |
| RIMKLA | 2.191922312 | 5.44E-05 |
| PRSS2 | 6.832691512 | 0.000789501 |
| HAPLN1 | 4.431861279 | 4.38E-07 |
| HPSE2 | 3.244909564 | 7.14E-05 |
| CRYGS | 2.869475863 | 0.000529462 |
| TNFRSF11B | 2.38428566 | 3.18E-05 |
| CDH10 | 2.4476235 | 1.64E-05 |
| C8orf88 | 2.028757001 | 8.20E-08 |
| CHODL | 3.171577692 | 4.01E-06 |
| DRD2 | 3.63315919 | 5.71E-06 |
| NDP | 2.72835258 | 0.000392266 |
| TSPAN10 | 2.014891408 | 4.02E-07 |
| PLAC1 | 3.130211613 | 3.05E-05 |
| CARD14 | 2.417342758 | 1.02E-05 |
| TNNT1 | 2.373085332 | 8.83E-07 |
| CHRNA1 | 5.21266873 | 0.000488053 |
| MMP13 | 3.152021007 | 2.35E-06 |
| AIM2 | 2.089325373 | 1.52E-05 |
| TFF2 | 2.157942233 | 2.84E-06 |
| SEZ6L | 4.997489385 | 0.000722979 |
| GNMT | -2.754559687 | 4.63E-12 |
| ARL9 | 2.027244623 | 0.000451311 |
| NEFH | 2.728572159 | 3.92E-08 |
| PRSS21 | 3.258499981 | 8.65E-06 |
| FABP5 | 2.166211106 | 2.08E-13 |
| DPF1 | 2.449703347 | 7.45E-07 |
| ISM2 | 3.710073425 | 4.71E-06 |
| KIF2C | 2.025524126 | 3.41E-18 |
| GABRA3 | 2.348178895 | 1.06E-07 |
| SV2A | 2.163209816 | 1.33E-07 |
| KCNV1 | 3.763996367 | 7.22E-10 |
| CALB2 | 3.631282551 | 1.10E-07 |
| FCGBP | 2.541581734 | 6.56E-13 |
| CST1 | 2.322746106 | 0.000473277 |
| IGDCC3 | 2.868390435 | 0.000166968 |
| SYT16 | 3.04264597 | 1.58E-06 |
| HSD17B13 | -3.450339223 | 1.86E-07 |
| SLC6A15 | 4.154942312 | 3.01E-05 |
| HAVCR1 | 2.638046648 | 6.52E-07 |
| CLEC2L | 2.303395904 | 1.07E-06 |
| TDRD5 | 2.718450484 | 1.72E-08 |
| SLC16A3 | 2.031573985 | 3.10E-11 |
| PSMA8 | 2.873759003 | 0.000452414 |
| HBQ1 | 2.817843531 | 9.70E-05 |
| MFAP2 | 2.172976609 | 3.13E-07 |
| SEC14L3 | -2.882991599 | 6.20E-09 |
| OPRPN | -3.70774052 | 1.35E-05 |
| CPA4 | 2.33745966 | 0.000717658 |
| SPATA16 | 2.16332711 | 1.35E-06 |
| CFHR3 | -2.418317634 | 3.95E-10 |
| TAT | -2.675439719 | 3.27E-11 |
| TEX15 | 2.679824454 | 2.39E-07 |
| LCTL | 2.183755595 | 0.000333097 |
| FZD2 | 2.018634009 | 3.96E-06 |
| LRP8 | 2.493352622 | 3.31E-12 |
| CADM2 | 2.435237193 | 1.39E-06 |
| PRAME | 2.136629078 | 1.35E-06 |
| VTCN1 | 2.020588344 | 1.51E-05 |
| MAGEA10 | 3.251486846 | 2.87E-07 |
| ERVMER34-1 | 2.658398061 | 7.17E-08 |
| TMEM63C | 2.494802529 | 2.89E-06 |
| MYADML2 | 2.068952651 | 1.21E-09 |
| TCF24 | 2.00731503 | 2.09E-09 |
| TGFB2 | 2.231772974 | 1.20E-05 |
| RFPL4B | 2.138582969 | 0.000990523 |
| TRPV5 | 2.215818814 | 5.37E-06 |
| PQLC2L | 2.829458968 | 1.01E-06 |
| ADAMTS20 | 2.005523867 | 1.39E-06 |
| CNDP1 | -3.46284981 | 8.32E-06 |
| HTR1F | 3.107003398 | 1.52E-05 |
| GAP43 | 2.30662076 | 5.15E-05 |
| CDH17 | 2.106814796 | 1.05E-07 |
| LRRIQ1 | 2.808159417 | 2.18E-08 |
| DNAJC5G | 2.167691332 | 5.44E-05 |
| ALX4 | 4.191050235 | 0.000325381 |
| MYBPC2 | 3.779639762 | 4.05E-05 |
| VWA2 | 2.325909922 | 0.000729965 |
| FKBP10 | 2.299672498 | 1.84E-06 |
| FNDC5 | -3.259152605 | 1.11E-07 |
| SLC28A3 | 2.384350196 | 0.000874194 |
| RCOR2 | 3.108788611 | 7.89E-11 |
| SERPINI1 | 2.037983026 | 9.01E-10 |
| FBP1 | -2.046877011 | 3.19E-14 |
| COLEC12 | 2.17731997 | 1.87E-06 |
| EFNA5 | 2.302376366 | 9.75E-07 |
| TMEM158 | 3.319191833 | 8.99E-06 |
| SORCS1 | 2.2141229 | 0.000293304 |
| APCDD1L | 4.514145148 | 6.21E-06 |
| CKMT1B | 2.945897279 | 4.81E-05 |
| LEMD1 | 2.804541631 | 0.000753055 |
| CTXN1 | 2.373336488 | 3.45E-06 |
| LIN28B | 2.578310001 | 1.12E-07 |
| RGS13 | 2.731025342 | 0.000110456 |
| ART5 | 2.177573739 | 0.000710083 |
| CYP3A4 | -2.651458305 | 2.64E-06 |
| AVPR1A | -2.268042915 | 0.000654702 |
| STRA6 | 2.010993826 | 7.92E-05 |
| CXorf66 | -3.570811851 | 2.14E-08 |
| SPOCD1 | 2.507528472 | 2.29E-07 |
| HECW1 | 2.078271249 | 5.85E-05 |
| PKIA | 2.79950557 | 1.10E-08 |
| DMBT1 | 3.835373695 | 0.000228828 |
| NLRP2 | 2.198394909 | 0.000338098 |
| IGSF23 | -2.206531518 | 1.67E-11 |
| FOXD1 | 3.506420619 | 0.000133887 |
| STK32A | 2.53845267 | 0.000124358 |
| CRYBB1 | 5.173329554 | 0.000720486 |
| ARL14 | 3.236434032 | 0.000684742 |
| CLPSL2 | 2.201766557 | 3.97E-05 |
| SDS | -2.651094549 | 5.90E-05 |
| CHGA | 5.639247399 | 5.15E-06 |
| RASSF9 | 2.698172415 | 9.55E-06 |
| HAO2 | -2.499710895 | 1.10E-08 |
| ADGRE1 | 2.355042413 | 6.43E-06 |
| CEP55 | 2.0032649 | 1.42E-14 |
| IGF2BP3 | 2.18243215 | 5.12E-10 |
| SPACA7 | -2.742150197 | 2.66E-07 |
| CA9 | 2.087146115 | 2.52E-10 |
| STARD6 | 2.221272842 | 1.82E-11 |
| SLC30A8 | 2.758165146 | 1.17E-07 |
| TUBAL3 | 3.332530961 | 3.68E-06 |
| HS3ST5 | 2.120672545 | 4.08E-08 |
| MFSD2A | -2.130714783 | 4.61E-05 |
| NEFL | 3.193605871 | 2.65E-05 |
| ISL2 | 2.410420835 | 5.48E-10 |
| MYBPC3 | 2.158299552 | 1.08E-08 |
| PRKCG | 2.654818215 | 1.94E-06 |
| ADGRB2 | 2.922859543 | 9.90E-06 |
| SHOX2 | 2.642597548 | 3.92E-08 |
| OLFM4 | 3.995053681 | 4.76E-06 |
| NTS | 4.276975113 | 2.21E-05 |
| SLC10A4 | 2.805578586 | 0.000837009 |
| ERP27 | 2.345536491 | 2.55E-05 |
| B3GNT7 | 2.065766676 | 0.000906824 |
| SNAP91 | 2.902272434 | 4.13E-05 |
| TERB2 | -2.551385497 | 0.000338337 |
| UCHL1 | 3.150956364 | 6.58E-07 |
| UPK1B | 2.346662036 | 0.00039697 |
| TRIM72 | 4.342441896 | 7.80E-06 |
| SLC10A1 | -3.086492379 | 1.51E-14 |
| TRIM67 | 2.212213252 | 4.34E-09 |
| SAGE1 | 2.723683778 | 4.82E-07 |
| PLEKHS1 | 2.904490506 | 5.86E-05 |
| CLDN6 | 3.595103689 | 0.000664297 |
| CAMKV | 2.010483853 | 0.000338036 |
| TRH | 4.045125454 | 4.85E-08 |
| HTR3A | 4.920937264 | 5.59E-05 |
| SEMA3A | 2.262441374 | 6.53E-07 |
| PGLYRP4 | 3.462650097 | 1.48E-05 |
| EPHA5 | 2.355658807 | 4.54E-07 |
| RHOV | 2.682399634 | 1.11E-05 |
| SPATA17 | 2.739728576 | 2.24E-12 |
| DDN | 2.636925054 | 1.01E-13 |
| PRAMEF15 | -2.258102385 | 0.000200016 |
| RGS17 | 2.31935026 | 3.21E-09 |
| TUBB3 | 2.507043279 | 7.82E-06 |
| CYP2A7 | -4.410685685 | 1.32E-08 |
| MYO3A | 3.091193269 | 6.04E-05 |
| CDK5R2 | 3.11572682 | 2.60E-10 |
| OPRD1 | 2.55399562 | 9.65E-08 |
| SOHLH2 | 3.15399614 | 9.60E-06 |
| TLR10 | 2.188390929 | 4.80E-05 |
| PTPRQ | 2.556830775 | 0.000239413 |
| GRIP1 | 2.188044853 | 0.000261638 |
| LHFPL5 | 2.867478384 | 8.27E-05 |
| L1CAM | 2.509509212 | 1.08E-05 |
| IGLON5 | 2.072369487 | 2.01E-06 |
| NHLH2 | 3.632357723 | 1.81E-08 |
| HMCN2 | -2.299716289 | 8.69E-08 |
| UNC13C | 2.83214812 | 0.00043674 |
| CCDC177 | -2.902696868 | 0.000202134 |
| PRDM9 | 2.161195095 | 0.000626693 |
| CUZD1 | 5.078183411 | 4.58E-06 |
| LHFPL3 | 2.303393402 | 6.51E-09 |
| GCNT1 | 2.272355728 | 4.95E-06 |
| CYP2B6 | -2.077498361 | 4.38E-08 |
| HOXC13 | 2.370736935 | 8.33E-05 |
| ACTL8 | 2.823351091 | 1.21E-08 |
| ST6GALNAC5 | 2.580522769 | 7.30E-09 |
| ATP6V1FNB | 2.05183649 | 5.14E-05 |
| UGT1A10 | 2.521674897 | 1.37E-05 |
| OTOGL | 2.346641338 | 2.42E-05 |
| ADCY1 | -2.086090655 | 0.000316299 |
| GLYAT | -2.097737319 | 3.98E-09 |
| NDST4 | 2.594061959 | 1.30E-09 |
| ANO4 | 2.190845206 | 6.98E-05 |
| FABP7 | 8.05774769 | 0.000270176 |
| ARMC3 | 3.208100472 | 1.21E-11 |
| NFE4 | 4.876541314 | 3.20E-05 |
| GLYATL3 | -2.361887184 | 0.000198713 |
| SH2D5 | 2.152496824 | 7.37E-06 |

Note: HCC, hepatocellular carcinoma.

Table S3. Association of the 127 cluster-related DEGs with the prognosis of HCC patients.

| Gene | HR | HR.95L | HR.95H | P |
| --- | --- | --- | --- | --- |
| BOLL | 66403.61842 | 222.1987651 | 19844577.16 | 0.000134526 |
| DRP2 | 3.393787075 | 1.221352868 | 9.430354658 | 0.019106578 |
| FIBCD1 | 1.201418888 | 1.017640594 | 1.418386171 | 0.030279838 |
| G6PD | 1.012815378 | 1.008712945 | 1.016934494 | 7.79E-10 |
| TTC36 | 0.990586633 | 0.981426551 | 0.999832211 | 0.046002998 |
| C7orf57 | 52.66050975 | 1.261633561 | 2198.046543 | 0.037339098 |
| PCK1 | 0.998226879 | 0.996591202 | 0.99986524 | 0.033918992 |
| FABP6 | 1.087079254 | 1.03484665 | 1.141948234 | 0.000889414 |
| HOXB8 | 1.189995065 | 1.002756558 | 1.412195456 | 0.046428211 |
| HOXD11 | 1.42653475 | 1.031508623 | 1.972839923 | 0.031753794 |
| TRIP13 | 1.142197446 | 1.090903993 | 1.195902678 | 1.42E-08 |
| CYP8B1 | 0.997036681 | 0.994759303 | 0.999319273 | 0.01097141 |
| ELFN1 | 0.956818523 | 0.926680366 | 0.987936854 | 0.006867672 |
| PPFIA4 | 2.063091948 | 1.284398563 | 3.313884419 | 0.002743667 |
| NEFM | 7.383984604 | 1.329626391 | 41.00642782 | 0.022273763 |
| STAC | 1.136394961 | 1.027880704 | 1.256365161 | 0.012525121 |
| GPR63 | 6.085349883 | 1.287775127 | 28.75617213 | 0.022657363 |
| CDC20 | 1.024872506 | 1.016463812 | 1.033350761 | 5.07E-09 |
| PLBD1 | 1.026843644 | 1.011167244 | 1.042763079 | 0.00073873 |
| DCHS2 | 44.96512843 | 2.005279492 | 1008.269811 | 0.016465008 |
| SLC2A1 | 1.060677084 | 1.033841048 | 1.088209719 | 6.63E-06 |
| DMRT2 | 1.761439511 | 1.165033639 | 2.663158423 | 0.007270428 |
| CCNJL | 1.636945867 | 1.39815106 | 1.916525223 | 9.02E-10 |
| PTPRN | 9.332301877 | 1.875581524 | 46.43458961 | 0.006368398 |
| PITX3 | 363.8727173 | 1.578787511 | 83863.94842 | 0.033629408 |
| IBSP | 1.016765089 | 1.00176327 | 1.031991567 | 0.028360914 |
| PKM | 1.002963986 | 1.000303517 | 1.00563153 | 0.028969905 |
| HEPACAM2 | 1.513000022 | 1.093078353 | 2.094240602 | 0.012541843 |
| FGF9 | 11.85093328 | 3.70028856 | 37.95504523 | 3.14E-05 |
| FBN2 | 2.413106693 | 1.226268585 | 4.748620311 | 0.010755555 |
| CALHM3 | 3.805724012 | 1.343016698 | 10.78432999 | 0.011906138 |
| PITX2 | 1.275866743 | 1.068425045 | 1.523584601 | 0.007123053 |
| SPIB | 1.016241711 | 1.000436578 | 1.032296537 | 0.043952693 |
| CFHR4 | 0.983373057 | 0.970314164 | 0.996607701 | 0.013965459 |
| PFN2 | 1.02363973 | 1.012006001 | 1.035407197 | 6.16E-05 |
| MPP2 | 2.840864973 | 1.874873886 | 4.304563552 | 8.46E-07 |
| MSC | 1.010073927 | 1.005388429 | 1.014781261 | 2.39E-05 |
| BCAT1 | 1.065405593 | 1.012428882 | 1.121154382 | 0.014906627 |
| MLF1 | 1.233539623 | 1.060021883 | 1.435460933 | 0.006656239 |
| GPR84 | 1.273499492 | 1.10177351 | 1.471991242 | 0.001070064 |
| KRT17 | 1.023577027 | 1.011335869 | 1.035966351 | 0.0001469 |
| EPHA6 | 1.825371687 | 1.188644927 | 2.803176727 | 0.005967689 |
| KIF3C | 1.112954547 | 1.034507946 | 1.197349744 | 0.004108825 |
| PKIB | 1.046305315 | 1.016568123 | 1.076912395 | 0.002091068 |
| NMU | 1.345867776 | 1.048889781 | 1.726930803 | 0.019532005 |
| DAW1 | 2119.15023 | 39.91103009 | 112520.2152 | 0.000157415 |
| CYP2C8 | 0.998174364 | 0.996447093 | 0.999904629 | 0.038649695 |
| PRR20G | 1.183500918 | 1.086811403 | 1.288792534 | 0.000106897 |
| RIPPLY2 | 1.27895766 | 1.115462862 | 1.466416096 | 0.000422259 |
| ANO1 | 0.981778534 | 0.967261761 | 0.996513176 | 0.015540525 |
| GYS2 | 0.981828417 | 0.968677662 | 0.995157706 | 0.007687626 |
| SPINK4 | 1.034730487 | 1.009631129 | 1.060453812 | 0.006430022 |
| CXCL5 | 1.008695008 | 1.001229547 | 1.016216133 | 0.02236169 |
| CTAG2 | 1.011673 | 1.003021007 | 1.020399624 | 0.008089603 |
| RFX6 | 1.672740007 | 1.117967099 | 2.502809905 | 0.012336811 |
| HMGA2 | 1.178400255 | 1.044738959 | 1.329161844 | 0.007529004 |
| NLRP6 | 0.849751352 | 0.7475313 | 0.965949333 | 0.01278347 |
| OR8A1 | 85.06178746 | 9.973131929 | 725.5000473 | 4.85E-05 |
| NR0B1 | 1.076622495 | 1.026456738 | 1.129239991 | 0.00242486 |
| ANKRD7 | 6.562062639 | 1.986851101 | 21.67281991 | 0.002027094 |
| ZNF280A | 11.21303049 | 3.306991907 | 38.02006666 | 0.000104542 |
| POU3F2 | 2.431600762 | 1.441817888 | 4.100852345 | 0.000861814 |
| ALX1 | 1.555968662 | 1.279752059 | 1.891802759 | 9.26E-06 |
| RIMKLA | 2.302992438 | 1.436296604 | 3.692673336 | 0.00053421 |
| TNFRSF11B | 1.015584827 | 1.001516977 | 1.029850282 | 0.029783717 |
| C8orf88 | 1.143790586 | 1.012411049 | 1.29221911 | 0.030919871 |
| CHODL | 1.046956354 | 1.015803002 | 1.079065139 | 0.002908108 |
| PLAC1 | 2.317680898 | 1.284127128 | 4.183109779 | 0.005270251 |
| FABP5 | 1.035108438 | 1.011766106 | 1.058989297 | 0.00302558 |
| ISM2 | 1.558434452 | 1.115218031 | 2.177796516 | 0.00935836 |
| KIF2C | 1.107275694 | 1.072814998 | 1.142843327 | 2.67E-10 |
| GABRA3 | 1.199491202 | 1.081139725 | 1.330798519 | 0.000599349 |
| SV2A | 1.167181876 | 1.011174745 | 1.347258265 | 0.034706429 |
| KCNV1 | 60.78396861 | 1.538948152 | 2400.789679 | 0.028538557 |
| CALB2 | 1.086183907 | 1.001489557 | 1.178040722 | 0.045944884 |
| FCGBP | 1.044867802 | 1.011592977 | 1.079237152 | 0.007860714 |
| SYT16 | 2868.399008 | 5.534854478 | 1486527.406 | 0.012542352 |
| HAVCR1 | 1.0533519 | 1.032254557 | 1.074880433 | 4.77E-07 |
| CLEC2L | 1.10183693 | 1.026567891 | 1.182624773 | 0.007225251 |
| SLC16A3 | 1.048105558 | 1.023234862 | 1.07358076 | 0.000125798 |
| PSMA8 | 1.394384386 | 1.027657113 | 1.891981083 | 0.032746322 |
| MFAP2 | 1.049985239 | 1.010574864 | 1.090932539 | 0.01245833 |
| CFHR3 | 0.990402802 | 0.984027358 | 0.996819553 | 0.003425308 |
| TAT | 0.998905571 | 0.997860291 | 0.999951946 | 0.0403717 |
| TEX15 | 3.351249687 | 1.951601305 | 5.754697147 | 1.17E-05 |
| LRP8 | 1.630299453 | 1.196200915 | 2.221931344 | 0.001974422 |
| PRAME | 1.024069884 | 1.008598562 | 1.039778527 | 0.002196429 |
| MAGEA10 | 1.134831504 | 1.036892954 | 1.24202073 | 0.006019878 |
| TCF24 | 4.501014276 | 1.1550363 | 17.53982062 | 0.03018507 |
| TGFB2 | 1.035077675 | 1.006785423 | 1.064164985 | 0.014759943 |
| RFPL4B | 1.165840648 | 1.070643844 | 1.269501919 | 0.000414669 |
| LRRIQ1 | 25.06902049 | 1.588614283 | 395.599986 | 0.022090767 |
| DNAJC5G | 3.118040294 | 1.745745056 | 5.569069344 | 0.000121667 |
| FKBP10 | 1.009648349 | 1.001909438 | 1.017447037 | 0.014449971 |
| RCOR2 | 1.188220977 | 1.092485363 | 1.292346001 | 5.73E-05 |
| EFNA5 | 1.083164908 | 1.014081364 | 1.156954716 | 0.017510113 |
| TMEM158 | 1.013248039 | 1.001100648 | 1.025542828 | 0.03245791 |
| LIN28B | 1.157528039 | 1.036845172 | 1.292257703 | 0.009212907 |
| ART5 | 1.21315567 | 1.005423479 | 1.463807748 | 0.043757327 |
| STRA6 | 1.290394515 | 1.026889789 | 1.621515787 | 0.028694995 |
| SPOCD1 | 1.213411729 | 1.034968298 | 1.422621376 | 0.01715015 |
| HECW1 | 3.552296941 | 1.305166689 | 9.668353988 | 0.013090272 |
| PKIA | 1.244955835 | 1.064157724 | 1.456471156 | 0.006206487 |
| CEP55 | 1.111571561 | 1.065462685 | 1.15967584 | 9.91E-07 |
| IGF2BP3 | 1.318404695 | 1.158327301 | 1.500604311 | 2.85E-05 |
| HS3ST5 | 4.286267493 | 1.55292585 | 11.83062863 | 0.004959621 |
| ISL2 | 1.381590542 | 1.058437049 | 1.803406664 | 0.01741914 |
| SLC10A4 | 1.486939231 | 1.073705145 | 2.059213638 | 0.016938465 |
| B3GNT7 | 1.017921476 | 1.004630767 | 1.031388014 | 0.008074139 |
| TERB2 | 0.41017684 | 0.207604835 | 0.810410027 | 0.010317 |
| CLDN6 | 1.14192628 | 1.069149958 | 1.219656436 | 7.81E-05 |
| PGLYRP4 | 1.976534894 | 1.055961413 | 3.69965241 | 0.033154659 |
| DDN | 9.859609362 | 3.215354399 | 30.23364915 | 6.26E-05 |
| RGS17 | 1.923833368 | 1.280271211 | 2.89089905 | 0.001638029 |
| SOHLH2 | 1.233676029 | 1.063911472 | 1.430529311 | 0.005433532 |
| TLR10 | 1.123464721 | 1.005682268 | 1.255041497 | 0.039375752 |
| PTPRQ | 2.222443077 | 1.256494897 | 3.930977549 | 0.006056925 |
| IGLON5 | 1.282484083 | 1.156020228 | 1.422782563 | 2.64E-06 |
| HMCN2 | 0.770371553 | 0.606116026 | 0.979139809 | 0.032985191 |
| UNC13C | 4236.152744 | 37.83758883 | 474263.5729 | 0.000521844 |
| PRDM9 | 3.179935821 | 1.611894472 | 6.273358462 | 0.000846528 |
| ST6GALNAC5 | 1.875438392 | 1.197113371 | 2.938125365 | 0.006042979 |
| UGT1A10 | 1.130090713 | 1.06349902 | 1.200852089 | 7.92E-05 |
| OTOGL | 25.16116003 | 2.196449546 | 288.2306015 | 0.009530742 |
| GLYAT | 0.991300204 | 0.983506675 | 0.99915549 | 0.030025129 |
| ARMC3 | 18.974802 | 3.02738399 | 118.9287887 | 0.001673223 |
| SH2D5 | 2.184201136 | 1.280259445 | 3.726381102 | 0.00415102 |

Note: HCC, hepatocellular carcinoma.
